# The Relationship Between a Mediterranean Diet and Frailty in Older Adults: NHANES 2007–2017

**DOI:** 10.3390/nu17020326

**Published:** 2025-01-17

**Authors:** Danae C. Gross, Jessica C. Dahringer, Paige Bramblett, Chang Sun, Hillary B. Spangler, David H. Lynch, John A. Batsis

**Affiliations:** 1 Department of Nutrition, Gillings School of Global Public Health, University of North Carolina at Chapel Hill, Chapel Hill, NC 27599, USApaige_bramblett@med.unc.edu (P.B.); cs123sun@gmail.com (C.S.); 2 School of Medicine, Virginia Commonwealth University, Richmond, VA 23298, USA; dahringerjc@vcu.edu; 3 Division of Geriatric Medicine, UNC School of Medicine, Chapel Hill, NC 27599, USA

**Keywords:** frailty, Mediterranean diet, aging

## Abstract

Background: Frailty is a geriatric syndrome of significant public health concern that causes vulnerability to physiologic stressors and an increased risk of mortality and hospitalizations. Dietary intake and quality are contributing factors to the development of frailty. The Mediterranean diet is known to be one of the healthiest eating patterns with promising health impacts for prevention. We evaluated the association between Mediterranean diet patterns and frailty status. Methods: We conducted a cross-sectional study using National Health and Nutrition Examination Survey data from 2007 to 2017. We included 7300 participants aged > 60 years who completed the first day of a 24 h diet recall and had full covariate data. We constructed an alternate Mediterranean diet (aMED) score based on the quantity of specific food-group intake and categorized participants to low-, moderate-, and high-adherence groups (aMED adherence scores of 0–2, 3–4, and 5–9, respectively). Using a modified Fried Frailty phenotype (weakness, low physical activity, exhaustion, slow walking speed, and weight loss), participants were categorized as robust (met no criteria), pre-frail (met one or two criteria), and frail (met three or more criteria). Logistic regression evaluated the association of frailty (prefrail/robust as referent) and aMED adherence. Results: Included participants were mainly female (54.5%) and non-Hispanic White (80.0%). The mean (SD) aMED score was 3.6 (1.6) with 45% of participants falling into moderate aMED adherence (26% low adherence, 30% high adherence). Frailty prevalence among participants was 7.1%, with most participants classified as robust (51.0%) or pre-frail (41.9%). Fully adjusted models showed significantly reduced odds of frailty with moderate-adherence and high-adherence groups (odds ratio (95%CI) of 0.71 (0.55, 0.92) and 0.52 (0.36, 0.75), respectively). Conclusions: Mediterranean diet adherence is associated with decreased odds of frailty in older adults. These findings suggest that adherence to a Mediterranean diet may play a critical role in mitigating frailty and its associated conditions. Future research should include longitudinal and interventional studies that can definitively determine the effect of a Mediterranean diet on frailty and what food components provide the greatest benefit.

## 1. Introduction

Frailty is a geriatric syndrome with significant public health implications that impacts over 15% of community-dwelling older adults in the United States (U.S.) and is present in up to 85% of older adults residing in long-term care facilities [1,2]. This syndrome is strongly associated with negative health outcomes due to physiologic vulnerability, which increases the risk of mortality, falls, and hospitalizations [3]. Frailty has traditionally been assessed using two models with different constructs—the Fried phenotype, which uses five components to indicate low physiologic reserve; and Rockwood’s Frailty index, which measures a person’s cumulative age-related health deficits [4,5]. While there are many factors that increase the development of frailty, inadequate nutritional quality is believed to be an important contributor with strong potential for practical clinical or educational interventions.

Data suggest that both undernutrition and overnutrition can contribute to an increased risk of frailty [6,7,8]. Appropriate nutritional interventions could delay the onset of frailty across the life course, particularly among older adults [9,10,11]. The Mediterranean diet is well established as one of the healthiest diets, providing beneficial effects among chronic diseases such as cardiovascular disease, various metabolic diseases, dementia, and cancer [12,13,14]. The Mediterranean diet focuses on a pattern consisting of a substantial intake of whole grains, fruits, vegetables, nuts, seeds, legumes, olives, and olive oil, moderate intake of dairy products, fish, and red wine, and restricted intake of meat and processed foods [15]. Monounsaturated fats, polyphenols, and polyunsaturated fats are key components of the Mediterranean diet, known to exert beneficial effects by reducing inflammation and oxidative stress, mechanisms that contribute to improved mitochondrial function, decreased cellular senescence, and enhanced muscle preservation—key factors in mitigating frailty and promoting healthful aging [12,16]. Thus, older populations whose intake parallels the Mediterranean diet could provide key insights into the development and mitigation of the risk of developing frailty.

With the increasing number of older adults in the U.S., understanding dietary patterns related to frailty are exceedingly relevant to potentially reduce the burden on patients, caregivers, and the health system. Although the Mediterranean diet has been well-studied, there is limited research exploring its relationship with frailty. To address this gap, we used data from the National Health and Examination Survey (NHANES) to understand adherence to a Mediterranean-diet-like pattern and frailty status in older adults.

## 2. Materials and Methods

### 2.1. Study Design

Data were obtained from NHANES 2007–2017, a cross-sectional survey conducted in non-institutionalized U.S. civilians for every two-year period by the National Center for Health Statistics of the Centers for Disease Control. NHANES was designed as a complex stratified survey that oversampled subpopulations, including Hispanic, non-Hispanic black, Asian, and low-income white populations, participants at or below certain threshold of poverty, those aged 80 years and older, and certain adolescent age groups. All participants provided written consent for the primary data collection. As the survey data are de-identified, the University of North Carolina at Chapel Hill’s Institutional Review Board determined this study to be exempt from review.

### 2.2. Study Population

The study cohort consisted of individuals aged 60 years or more who completed a 24 h diet recall and who were not missing covariate data from the 2007–2017 survey cycles (Figure S1). Of the 70,190 NHANES participants, 62,890 NHANES participants were excluded because they had not completed a dietary recall (*n* = 9459), were less than 60 years of age (*n* = 50,232), or were missing covariate data (*n* = 3109). The final cohort consisted of 7300 participants who completed at least one 24 h diet recall, were aged greater than 60 years, and had full covariate data.

### 2.3. Primary Outcome: Frailty Status

Our primary outcome was frailty status (robust, pre-frail, and frail). The Fried Frailty phenotype is a well-established construct used in clinical settings that uses both objective and self-reported measures to categorize frailty status [4]. Table 1 summarizes Fried Frailty phenotype indicators used to assess frailty status using self-reported NHANES data [9,17,18]. Weight and height were obtained at the NHANES Mobile Examination Center by trained health technicians using standard procedures. Weight was measured on an electronic digital scale, calibrated in kilograms. Participants whose self-reported current weight was at least 10 pounds lower than their reported weight a year ago were asked if that weight change was intentional. Height was measured standing on a vertical backboard of a stadiometer, with their body weight evenly distributed on both feet after deep inhalation. BMI was calculated using weight (kg) divided by height (m^2^) and categorized as follows: underweight (≤18.5 kg/m^2^), normal (18.5–24.9 kg/m^2^), overweight (25.0–29.9 kg/m^2^), and obese (≥30 kg/m^2^). Using the frailty indicators, participants were categorized as robust if they were without any indicators, pre-frail if they had one or two indicators, and frail with three or more indicators.

### 2.4. Primary Predictor: Mediterranean Diet Adherence

Mediterranean diet adherence was assessed using methods similar to those of Trichopoulou et al. [19] and adapted by Fung et al. [20], which utilized food frequency questionnaires to evaluate the consumption of vegetables, legumes, fruit and nuts, dairy, cereals, meat and meat products, fish, alcohol, and the ratio of monounsaturated to saturated fat. An intake above the median for each food group is given a point of 1; all others were given a 0. The exceptions to this were for meat and alcohol; 1 point was given if the intake was below the median meat intake, and 1 point was given if alcohol intake was between 5 and 15 g/day. We adapted this to develop an alternate Mediterranean diet (aMED) score to use with the NHANES 24 h recall of dietary intake.

The 24 h recall was conducted in person at the Mobile Examination Center through the “What We Eat in America” interview, which was led by the U.S. Department of Agriculture (USDA) and the U.S. Department of Health and Human Services [21]. The USDA used the automated multiple-pass method as a computerized system to collect dietary data during the interview. Each food and beverage consumed by the participant in the past 24 h was recorded, including a detailed description of the amount, setting, timing, and nutritional information about the food. Using the Food Pattern and Equivalents Database, dietary data were converted to the 37 USDA food pattern components and the following groups were utilized for our study: (1) fruit (whole or cut intact fruit and fruit juices) in cup equivalents; (2) whole grains (contains the bran, germ and endosperm) in cup equivalents; (3) legumes (beans and peas) in cup equivalents; (4) vegetables (dark green, red, orange, and other vegetables not including potatoes) in cup equivalents; (5) nuts and seeds (peanuts, tree nuts, and seeds, excluding coconuts) in oz. equivalents; (6) seafood (finfish, shellfish, and other seafood) in oz. equivalents; (7) red meat (beef, veal, pork, lamb, and game meat) and processed meat (frankfurters, sausages, corned beef, and luncheon meat that are made from beef, pork or poultry) in oz. equivalents; (8) alcohol in grams (g); and (9) a ratio of monosaturated fatty acids to saturated fatty acids in g [22]. An intake above the median for each food group was given a score of 1; all others received a score of 0. The exceptions to this were for meat and alcohol; one point was given if the intake was below the median meat intake, and one point was given if alcohol intake met the sex-specific recommendations—between 5 and 15 g/day (females) and 10–25 g/day (males) [22]. See Supplementary Table S2.6 for details.

Data from the 2020–2025 Dietary Guidelines for Americans suggest that food-group intake varies across age groups; therefore, we based participants’ aMED scores on the median intake values of adults ages 18 years or older to provide a more accurate representation of participant aMED scores [22].

### 2.5. Covariates

Self-reported demographic, health, and nutrition information were collected by interviewers using a computer-assisted software system. Age is presented in years and in age groups (60–69, 70–79, or 80–89 years), and sex reported as female or male. We categorized race and ethnicity as self-reported non-Hispanic White, non-Hispanic Black, Hispanic, and other (includes multi-racial); education level (less than high school, high school, some college or 2-year degree, and college graduate or above); and smoking status (nonsmoker, or history of smoking 100 cigarettes in their lifetime). Self-reported medical conditions include arthritis, diabetes, cardiovascular disease, stroke, chronic obstructive pulmonary disease (COPD), hypertension, cancer, kidney disease, and depression. One point was allocated for each positive history, which was summed to create a medical comorbidity score (range 0–9). Self-reported polypharmacy, taking five or more medications, was included because of its relationship with an increased risk of geriatric syndromes such as frailty and falls [23].

### 2.6. Statistical Analyses

All data were downloaded, aggregated, and analyzed using the NHANES analytical guidelines accounting for primary sampling units, strata, and weights. Descriptive statistics for continuous and categorical variables are represented as the mean (standard deviation) using Kruskal–Wallis tests for statistical significance and unweighted counts (weighted percent) using chi-squared tests for statistical significance. Univariate (model 1) and multivariate binomial logistic regression analyses evaluated the association of frailty (robust and pre-frail as the referent group) with aMED diet adherence (predictor), both as a continuous variable (score of 0–9) and a categorical variable. The categories of aMED adherence were low (score of 0–2, referent), moderate (score of 3–4), and high (score of 5–9). Multivariate models adjusted for relevant covariates. Model 2 included sex, age (continuous), race/ethnicity, and education; model 3 was as per model 2 plus smoking status; and model 4 was as per model 3 plus polypharmacy and comorbidities score. Sensitivity analyses were conducted to assess the robustness of the association between aMED and frailty status. We evaluated the predictor as a 4-category variable: low (score of 0–2, referent), low moderate (score of 3), high moderate (score of 4), and high (score of 5–9); and tertiles and quartiles of the aMED adherence score were used. We also used pre-frail and frail as the outcomes (robust as the referent) for logistic regression. This approach was carried out for simplicity of interpretation of the odds ratios and 95% confidence intervals (95%CI). Additionally, all analyses were repeated for frailty definitions 2 and 3. Statistical analyses were performed using R version 4.0.4 (www.r-project.org) with the dplyr (v1.1.4), tidyverse (v1.3.1), and gtsummary (v1.5.2) packages [24,25,26]. A *p*-value of <0.05 was considered statistically significant.

## 3. Results

Baseline characteristics of the study population are compared by aMED adherence status in Table 2. The 7300 individuals were categorized into groups of low (N = 1966), moderate (N = 3323), and high (N = 2011) adherence. The mean age of participants was 69.9 ± 6.7 years (54.5% female), with 80% being non-Hispanic White, and 51.5% of participants were aged between 60 and 69 years, 32.5% between 70 and 79 years, and 15.9% between 80 and 89 years. Significant trends were observed across BMI categories, education, physical activity, smoking status, and medical conditions by aMED adherence status (see Table S1). Similar findings were observed in sensitivity analyses using adherence categories based on four categories (Table S1.1), tertiles (Table S1.2), quartiles (Table S1.3), and when stratified by sex (Table S1.5). Compared to older adults excluded from our analysis, our study population was younger, more educated and physically active, with a higher income. However, we noted that our study population had more medical conditions and a higher BMI; see Table S1.4 for more details.

Prevalence of the frailty indicators across aMED adherence can be found in Table 3. Frailty prevalence was 7.1% (*n* = 610), with the highest prevalence in the low-adherence group (10.6%) while those classified as robust made up 51.0% of participants, with the highest prevalence in the high-adherence group (56.3%). Similar findings were observed in our sensitivity analyses of frailty definitions 2 and 3 (Table S2.1), as well as aMED adherence categories based on four categories (Table S2.2), tertiles (Table S2.3), and quartiles (Table S2.4). Sensitivity analyses of individual indicator prevalence across each definition of frailty can be found in Supplementary Table S2.5.

We observed significant differences in aMED adherence across frailty status (Table 4). The mean aMED adherence score was higher in the robust group (3.8 ± 1.6) compared to the pre-frail (3.1 ± 1.6) and frail (3.1 ± 1.5) groups (*p* < 0.001). Individuals with higher aMED adherence were classified as robust (32.9%) compared to pre-frail and frail groups (28.1% and 17.9%, respectively). Frail individuals had a significantly lower intake of vegetables (42.5% vs. 54.7% robust, 50.3% pre-frail, *p* < 0.001) and nuts (32.4% vs. 48.0% robust, 43.9% pre-frail, *p* < 0.001), and a lower MFA to SFA ratio (41.9% vs. 52.7% robust, 52.9% pre-frail, *p* = 0.006). Whole grain (54.1% vs. 62.1% robust, *p* = 0.009) and fish (14.1% vs. 20.0% robust, *p* = 0.016) intake were also lower in the frail group. Alcohol guideline adherence was lowest in the frail group (3.6% vs. 8.8% robust, *p* < 0.001). No significant differences were found for fruit, legumes, or red meat intake. Similar findings were observed in our sensitivity analyses (Tables S3.1 and S3.2).

Multivariate analyses are presented in Table 5. In the unadjusted model, higher adherence scores are associated with lower odds of frailty with an OR (95%CI) of 0.79 (0.73, 0.84), which remained significant in the fully adjusted model [0.84 (0.78, 0.91)]. Similar results were observed across aMED adherence groups. The unadjusted ORs (95%CI) for the moderate- and high-adherence groups were 0.64 (0.50, 0.82) and 0.38 (0.26, 0.54), respectively, which remained significant in the in the fully adjusted model [0.71 (0.55, 0.92) and 0.52 (0.36, 0.75), respectively]. Similar findings were observed in sensitivity analyses (Tables S4.1–S5.4).

## 4. Discussion

Our study demonstrates an association between aMED adherence and frailty among older adults, as assessed through multiple frailty indicators and overall frailty status. These findings are consistent across various sensitivity analyses. Participants’ baseline characteristics revealed significant differences across aMED adherence groups in terms of BMI, education level, and race/ethnicity. Those with higher adherence to aMED were more likely to have a healthier BMI and higher education levels. This aligns with previous research indicating that higher diet quality is often associated with more favorable socio-demographic factors and better health outcomes [27,28,29,30].

Overall, weakness was the most prevalent frailty indicator among participants, followed by low physical activity. There is a known overlap in pathophysiology between sarcopenia and frailty, as sarcopenia and a sedentary lifestyle can lead to decreased muscle strength and mobility which can elicit or exacerbate the onset of weakness [31]. However, low physical activity was not significantly different between the aMED adherence groups (*p* = 0.4). Self-reported measures can be prone to inaccuracies and might not capture the full extent of an individual’s activity levels. Additionally, physical activity levels may be uniformly low in this population, regardless of diet. Despite this, the prevalence of frailty varied across aMED adherence levels. The low-adherence group had the highest prevalence of frailty (10.6%) and positive frailty indicators, such as exhaustion (21.8%), slow walking speed (12.3%), weakness (27.6%), and unintentional weight loss (10.0). Conversely, the high-aMED-adherence group had the lowest prevalence.

The observed differences in aMED adherence across frailty groups have important clinical implications. Higher adherence to a Mediterranean diet, particularly among the robust group, underscores the potential role of Mediterranean dietary patterns in maintaining physical robustness and delaying or mitigating frailty progression. We evaluated each component of an aMED (Table 4) and found vegetables, whole grains, nuts, fish, MFA:SFA, and alcohol were significantly lower in the frail group, which aligns with established evidence linking poor diet quality to adverse health outcomes, such as inflammation, muscle loss, and functional decline. However, there was no significant difference with fruit, legumes, and red meat. While fruits and legumes are nutrient dense, their specific impact on frailty may not be as pronounced as other components of the Mediterranean diet, such as vegetables, nuts, and fish, which provide unique nutrients and bioactive compounds known to influence muscle health and physical function [32,33,34,35]. It is also important to note that the U.S. Dietary Guidelines for Americans 2020–2025 reported that older adults aged 60+ years do not consume enough fruits, vegetables, and whole grains [22]. Red meat consumption might not vary significantly as it is a common dietary component among a wide range of people, regardless of frailty status or diet quality. These findings suggest that tailored dietary interventions focusing on Mediterranean diet patterns could serve as a valuable strategy for frailty prevention.

While recent systematic reviews have shown a significant association between the Mediterranean diet and frailty status in older adults, few studies have explored this association in the U.S. population, and there is little data evaluating individual Mediterranean-diet food-group components and their relationship with frailty status [36,37,38]. This study provides a greater understanding of the diet’s role in the U.S. population of older adults by utilizing an expansive database, analyzing aMED components among participants with varying degrees of frailty, and evaluating frailty status using three adaptions of the frailty status. Self-reported frailty indicators are practical options for clinical practice and research, enabling providers and individuals to more easily assess frailty status. Additionally, this study highlights key food components that could inform future research on dietary interventions to prevent or treat frailty.

This study has several limitations. First, the self-reported 24 h diet recall may not accurately reflect habitual dietary patterns and is prone to underreporting and overreporting, increasing the risk of misclassification. Future studies should incorporate multiple 24 h diet recalls or food frequency questionnaires to more accurately capture habitual dietary intake. Second, as a cross-sectional study, NHANES cannot establish causal inferences about the relationship between Mediterranean diet adherence and frailty. Additionally, we used frailty indicators based on self-reported measures instead of objective measures used in the Fried Frailty phenotype. Additionally, it is possible that there is residual confounding not included in this analysis, like food availability, social support, or other systemic factors. Despite these limitations, our study has notable strengths. Adapting the Fried Frailty phenotype is supported by similar methods in other epidemiologic studies [18,39,40]. We included three definitions of frailty to capture all possible frail and pre-frail individuals. NHANES’s oversampling of underrepresented populations improves the reliability and precision of health status estimates, and the large sample size enhances generalizability to the U.S. population. Sensitivity analyses for frailty indicators and aMED adherence demonstrated consistent findings, further supporting our results.

## 5. Conclusions

This study utilizes alternative indicators of frailty status and Mediterranean diet adherence that clinicians and researchers can easily incorporate into clinical and research settings to determine a patient’s frailty status based on their dietary intake. The use of a modified Fried Frailty phenotype with self-reported measures, as demonstrated in this study, could be particularly useful for rural populations or clinics lacking the resources to obtain objective measures, as well as for telehealth applications. The Mediterranean diet is oftentimes used for counselling patients on reducing the risk of cardiovascular events and stroke, and its strong association with reduced frailty can be emphasized in preventative education for aging adults. The findings from this study could inform future interventional studies aimed at identifying which specific components of the Mediterranean diet have the most significant impact on reducing frailty status. Future research should explore the combined effects of the Mediterranean diet and physical activity in preventing frailty, as studies have shown that these complementary interventions promote healthy aging. The Mediterranean diet is known to reduce inflammation and oxidative stress, while physical activity enhances muscle strength, cardiovascular health, and bone density—key factors in frailty prevention. Studies have demonstrated that combining resistance exercise and/or aerobic exercise with the Mediterranean diet improves muscle mass, strength, and overall physical function. Future longitudinal studies could assess the synergistic effects of these interventions on frailty outcomes, particularly in older adults at risk of sarcopenia and other age-related conditions, providing valuable insight into effective strategies for frailty prevention.

## Figures and Tables

**Table 1:** Comparison of Indicators used for Fried Frailty Phenotype and Modified Fried Frailty Phenotype.

Indicator	Fried Frailty Phenotype [4]	Modified Fried Frailty Phenotype [17]
Weakness	Direct measure: grip strength—lowest 20% (adjusted for gender, BMI)	Self-reported: difficulty with lifting or carrying something as heavy as 10 lbs
Low Physical Activity	Self-reported: lowest 20% of calories expended per week (adjusted for gender)	Self-reported: highest 20% of minutes per day of sedentary activity
Exhaustion	Self-reported: “moderate amount of time” or “most of the time” in the last week to at least one of the following statements: (a) I felt that everything I did was an effort; (b) I could not get going	Self-reported: feelings of tiredness or having little energy over the past two weeks for “more than half the days” or “nearly every day”
Slow Walking Speed	Direct measure: 15 ft walk time—slowest 20% (by gender and height)	Self-reported: difficulty walking between rooms on the same floor
Weight Indicator	Self-reported: unintentional weight loss of ≥10 lbs in prior year (not due to diet and exercise), OR direct measure: at follow-up, ≥5% of body weight gained in prior year (weight is a direct measure, self-reported unintentionally)	Definition 1 (self-reported): unintentional weight loss ≥10 lbs in the previous year, OR definition 2 (self-reported): weight loss ≥10 lbs in the previous year, OR definition 3 (direct measure): low BMI defined as ≤18.5 kg/m^2^

Abbreviations: BMI (body mass index); ft (foot); kg (kilogram); lb (pound); m (meter).

**Table 2:** Baseline Characteristics of Study Population by Mediterranean Diet Adherence.

Variable	Overall ^1^ N = 7300 (100)	Low ^1^ N = 1966 (26)	Moderate ^1^ N = 3323 (45)	High ^1^ N = 2011 (30)	*p*-Value ^2^
Age (years)	69.9 (6.7)	69.3 (6.7)	70.1 (6.8)	69.9 (6.7)	0.041
Age Category (years)					0.6
60–69	3529 (51.5)	964 (53.7)	1609 (50.6)	956 (51.2)	
70–79	2451 (32.5)	670 (31.7)	1109 (32.9)	672 (32.7)	
80–89	1320 (15.9)	332 (14.6)	605 (16.5)	383 (16.2)	
Sex (Female)	3697 (54.5)	901 (50.9)	1699 (53.8)	1097 (58.6)	0.001
Race/Ethnicity					<0.001
Non-Hispanic White	3834 (80.0)	1047 (80.5)	1705 (79.2)	1082 (80.7)	
Non-Hispanic Black	1509 (8.1)	451 (9.5)	700 (8.5)	358 (6.3)	
Hispanic	1449 (6.9)	387 (7.2)	719 (7.6)	343 (5.4)	
Other	508 (5.1)	81 (2.8)	199 (4.7)	228 (7.5)	
BMI (kg/m^2^)	29.6 (6.4)	30.8 (7.3)	29.8 (6.0)	28.2 (5.9)	<0.001
BMI category (kg/m^2^)					<0.001
<18.5	80 (0.9)	23 (0.8)	36 (0.9)	21 (0.9)	
18.5–24.9	1579 (22.1)	364 (17.9)	661 (18.6)	554 (30.8)	
25–29.9	2624 (35.8)	689 (33.1)	1196 (37.2)	739 (36.0)	
30–34.9	1788 (24.5)	499 (26.1)	838 (26.4)	451 (20.4)	
35–39.9	757 (10.5)	234 (12.7)	359 (10.9)	164 (8.1)	
≥40	472 (6.2)	157 (9.4)	233 (5.9)	82 (3.8)	
Education Level					<0.001
≤12th grade	1964 (16.4)	656 (22.2)	937 (17.3)	371 (10.0)	
High school graduate/GED	1792 (25.5)	559 (30.5)	832 (26.4)	401 (19.9)	
Some college or AA degree	1997 (29.7)	512 (30.1)	937 (31.1)	548 (27.1)	
College graduate or above	1547 (28.5)	239 (17.2)	617 (25.2)	691 (43.1)	
Relationship Status ^3^	4284 (65.2)	1074 (60.2)	1942 (65.3)	1268 (69.3)	<0.001
Income Poverty Level	3.1 (1.6)	2.8 (1.5)	3.1 (1.6)	3.5 (1.5)	<0.001
Mediterranean Diet Score	3.6 (1.6)	1.6 (0.6)	3.5 (0.5)	5.6 (0.8)	<0.001
Physically Active	4075 (62.5)	946 (53.1)	1810 (60.0)	1319 (74.1)	<0.001
Smoking Status ^4^	3822 (50.9)	1191 (58.7)	1704 (50.3)	927 (45.2)	<0.001
Polypharmacy ^5^	3210 (41.7)	957 (46.8)	1528 (43.6)	725 (34.4)	<0.001
Lives Alone	1906 (24.0)	518 (25.7)	868 (23.8)	520 (22.8)	0.3
Medical Conditions ^6^	2.3 (1.4)	2.5 (1.5)	2.3 (1.4)	2.1 (1.4)	<0.001
Arthritis	3980 (55.3)	1125 (59.0)	1789 (54.6)	1066 (53.2)	0.069
Cancer	1688 (26.4)	469 (27.2)	735 (25.7)	484 (26.8)	0.7
Stroke	652 (7.7)	216 (9.5)	305 (8.0)	131 (6.0)	0.007
Pulmonary Disease	1526 (21.8)	465 (25.5)	716 (22.5)	345 (17.8)	<0.001
Cardiovascular Disease	1295 (17.0)	407 (17.7)	578 (17.8)	310 (15.2)	0.2
Hypertension	5033 (65.0)	1395 (65.9)	2303 (66.7)	1335 (61.6)	0.044
Diabetes	2036 (23.0)	580 (24.4)	964 (25.1)	492 (18.5)	<0.001
Kidney Disease	479 (5.0)	185 (7.9)	205 (4.6)	89 (3.1)	<0.001
Depression	591 (6.9)	218 (10.1)	266 (6.8)	107 (4.2)	<0.001

^1^ Continuous aMED adherence score (range 0 to 9) used for aMED adherence categories: low, 0–2; moderate, 3–4; and high, 5–9. Descriptive statistics presented as unweighted N (weighted %); mean (SD). ^2^ Chi-squared test with Rao and Scott’s second-order correction; Kruskal–Wallis rank-sum test for complex survey samples. ^3^ Married or lives with partner (vs. widowed, separated, divorced, or single). ^4^ Current smoker or history of smoking 100 or more cigarettes during lifetime. ^5^ Taking 5 or more medications. ^6^ Summary score of comorbidities using self-reported history of arthritis, cancer, stroke, pulmonary disease, cardiovascular disease, hypertension, diabetes, kidney disease, and depression (range 0 to 9). Abbreviations: AA (Associate of Arts), aMED (alternate Mediterranean diet); BMI (body mass index), GED (General Education Development), kg (kilogram), m (meter), N (number); SD (standard deviation).

**Table 3:** Frailty Indicators and Frailty Status by Mediterranean Diet Adherence.

	Overall ^1^ N = 7300 (100)	Low ^1^ N = 1966 (26)	Moderate ^1^ N = 3323 (45)	High ^1^ N = 2011 (30)	*p*-Value ^2^
**Frailty Indicators ^3^**					
Low Physical Activity	1368 (21.1)	381 (22.3)	625 (21.3)	362 (19.7)	0.4
Exhaustion	1330 (16.9)	433 (21.8)	605 (16.6)	292 (13.1)	<0.001
Slow Walking Speed	780 (8.5)	279 (12.3)	340 (7.9)	161 (6.1)	<0.001
Weakness	1993 (22.9)	617 (27.6)	921 (22.9)	455 (18.8)	<0.001
Unintentional Weight Loss	693 (8.0)	254 (10.0)	293 (7.7)	146 (6.7)	0.034
**Frailty Status**					<0.001
Robust	3517 (51.0)	839 (45.1)	1592 (50.8)	1086 (56.3)	
Pre-frail	3173 (41.9)	907 (44.3)	1455 (42.2)	811 (39.4)	
Frail	610 (7.1)	220 (10.6)	276 (7.0)	114 (4.3)	

^1^ Continuous aMED adherence score (range 0 to 9) used for aMED adherence categories: low, 0–2; moderate, 3–4; and high, 5–9. Descriptive statistics presented as unweighted N (weighted %). ^2^ Chi-squared test with Rao and Scott’s second-order correction. ^3^ Modified Fried Frailty phenotype is based on 5 indicators: (a) self-reported weakness: difficulty with lifting or carrying something as heavy as 10 pounds; (b) self-reported low physical activity: top quintile of minutes of sedentary activity; (c) self-reported exhaustion: feelings of tiredness or having little energy over the past two weeks for “more than half the days” or “nearly every day”; (d) self-reported slow walking speed: difficulty walking between rooms on the same floor; and (e) self-reported unintentional weight loss: ≥10 lbs in the previous year; Abbreviations: aMED (alternate Mediterranean diet); lb (pound); N (number).

**Table 4:** Components of Mediterranean Diet by Frailty Status.

	Overall ^1^ N = 7300 (100)	Robust ^1^ N = 3517 (51)	Pre-Frail ^1^ N = 3173 (42)	Frail ^1^ N = 610 (7.1)	*p*-Value ^2^
**aMED Score ^3^**	3.6 (1.6)	3.8 (1.6)	3.6 (1.6)	3.1 (1.5)	<0.001
**aMED Adherence Category ^3^**					<0.001
Low	1966 (25.5)	839 (22.6)	907 (27.0)	220 (38.0)	
Moderate	3323 (44.7)	1592 (44.5)	1455 (45.0)	276 (44.1)	
High	2011 (29.8)	1086 (32.9)	811 (28.1)	114 (17.9)	
**Adherence to aMED Food Group ^4^**					
Fruit	4306 (58.8)	2148 (60.5)	1817 (57.3)	341 (56.0)	0.14
Vegetables	3480 (52.0)	1772 (54.7)	1471 (50.3)	237 (42.5)	<0.001
Whole Grains	4117 (59.8)	2048 (62.1)	1751 (58.1)	318 (54.1)	0.009
Legumes	1452 (18.1)	735 (19.5)	609 (17.0)	108 (15.2)	0.076
Nuts	2783 (45.2)	1446 (48.0)	1158 (43.9)	179 (32.4)	<0.001
Fish	1387 (18.3)	723 (20.0)	572 (17.0)	92 (14.1)	0.016
Red Meat	3910 (51.7)	1848 (51.1)	1740 (52.9)	322 (48.7)	0.4
MFA:SFA	4017 (52.0)	1967 (52.7)	1760 (52.9)	290 (41.9)	0.006
Alcohol	475 (7.3)	289 (8.8)	164 (6.1)	22 (3.6)	<0.001
**Serving of aMED Food Group**					
Fruit (cup)	1.0 (1.2)	1.1 (1.2)	1.0 (1.2)	1.0 (1.3)	0.075
Vegetables (cup)	1.2 (1.2)	1.3 (1.3)	1.1 (1.1)	1.0 (1.0)	<0.001
Whole Grains (cup)	1.0 (1.3)	1.1 (1.4)	1.0 (1.3)	0.8 (1.2)	0.019
Legumes (cup)	0.1 (0.3)	0.1 (0.3)	0.1 (0.3)	0.1 (0.3)	0.090
Nuts (cup)	0.8 (2.1)	0.9 (2.0)	0.8 (2.2)	0.6 (1.6)	<0.001
Fish (oz)	0.7 (2.0)	0.7 (2.1)	0.6 (2.0)	0.5 (1.9)	0.012
Red Meat (oz)	2.4 (2.7)	2.3 (2.5)	2.4 (2.8)	2.5 (2.7)	0.5
MFA:SFA	1.2 (0.4)	1.2 (0.4)	1.2 (0.4)	1.1 (0.5)	<0.001
Alcohol (gm)	6.6 (17.7)	7.1 (16.6)	6.4 (19.0)	3.7 (16.9)	<0.001

^1^ Modified Fried Frailty phenotype is based on 5 indicators (a) self-reported weakness: difficulty with lifting or carrying something as heavy as 10 pounds; (b) self-reported low physical activity: top quintile of minutes of sedentary activity; (c) self-reported exhaustion: feelings of tiredness or having little energy over the past two weeks for “more than half the days” or “nearly every day”; (d) self-reported slow walking speed: difficulty walking between rooms on the same floor; and (e) self-reported unintentional weight loss: ≥10 lbs in the previous year; robust: 0 indicators, pre-frail: 1 to 2 indicators, frail: 3 or more indicators. Descriptive statistics presented as unweighted N (weighted %); mean (SD); ^2^ Kruskal–Wallis rank-sum test for complex survey samples; chi-squared test with Rao and Scott’s second-order correction; ^3^ continuous aMED adherence score (range 0 to 9) used for aMED adherence categories: low, 0–2; moderate, 3–4; and high, 5–9; ^4^ adherence to aMED food groups: intake ≥ median for fruit, vegetables, whole grains, legumes, nuts, and fish; <median for red meat and MFA:SFA; and within the sex-specific recommendations for alcohol; Abbreviations: aMED (alternate Mediterranean diet); gm (gram); lb (pound); MFA:SFA (monosaturated fat to saturated fat ratio); N (number); oz (ounce); SD (standard deviation).

**Table 5:** Univariate and Multivariate Logistic Regression of Frailty by Mediterranean Diet Adherence.

	aMED Score ^1^	Low ^1^	Moderate ^1^	High ^1^
Frail vs. Robust/Pre-frail ^2^	N = 610 (7.1%)	N = 220 (10.6%)	N = 276 (7.0%)	N = 114 (4.3%)
Model 1	0.79 (0.73, 0.84)	Ref.	0.64 (0.50, 0.82)	0.38 (0.26, 0.54)
Model 2	0.81 (0.75, 0.87)	Ref.	0.64 (0.50, 0.82)	0.42 (0.29, 0.61)
Model 3	0.81 (0.75, 0.87)	Ref.	0.65 (0.51, 0.84)	0.43 (0.30, 0.62)
Model 4	0.84 (0.78, 0.91)	Ref.	0.71 (0.55, 0.92)	0.52 (0.36, 0.75)

^1^ Continuous aMED adherence score (range 0 to 9) used for aMED adherence categories: low, 0–2 (referent); moderate, 3–4; and high, 5–9. Descriptive statistics presented as unweighted N (% weighted); total unweighted N = 7300; OR (95%CI). ^2^ Frail: three or more indicators; robust/pre-frail: two or less indicators. Modified Fried Frailty phenotype is based on 5 indicators (a) self-reported weakness: difficulty with lifting or carrying something as heavy as 10 pounds; (b) self-reported low physical activity: top quintile of minutes of sedentary activity; (c) self-reported exhaustion: feelings of tiredness or having little energy over the past two weeks for “more than half the days” or “nearly every day”; (d) self-reported slow walking speed: difficulty walking between rooms on the same floor; and (e) self-reported unintentional weight loss: ≥10 lbs in the previous year. Model 1: unadjusted; model 2: adjusted for sex, age, race, and education; model 3: model 2 + smoking status; model 4: model 3 + polypharmacy and medical comorbidities score. Abbreviations: aMED (alternate Mediterranean diet); CI (confidence interval); lb (pound); N (number); OR (odds ratio).

## Data Availability

The original data presented in the study are openly available at the National Health and Nutrition Examination Survey at https://wwwn.cdc.gov/nchs/nhanes/.

## Supplementary Materials

The supporting information can be downloaded at: https://www.mdpi.com/article/10.3390/nu17020326/s1.

## Abbreviations

The following abbreviations are used in this manuscript:

aMED	Alternate Mediterranean Diet
BMI	Body Mass Index
COPD	Chronic Obstructive Pulmonary Disease
MFA	Monosaturated Fatty Acids
NHANES	National Health and Nutrition Examination Survey
OR (95%CI)	Odds Ratio (95% Confidence Interval)
SFA	Saturated Fatty Acids
U.S.	United States
USDA	United States Department of Agriculture
