# Supplementary material for: The Relationship Between a Mediterranean Diet and Frailty in Older Adults: NHANES 2007–2017
Source: Nutrients. 2025 Jan 17;17(2):326. doi: 10.3390/nu17020326 (PMC11767853; doi:10.3390/nu17020326)
Supplement: Supplementary file 1 [file nutrients-17-00326-s001.zip › nutrients-3412944-supplementary.pdf]

Supplementary Tables	
Figure S1	Participant Inclusion/Exclusion
Table S1	Baseline Characteristics of Study Population by Mediterranean Diet Adherence (3 categories) – with confidence intervals
Table S1.1	Baseline Characteristics of Study Population by Mediterranean Diet Adherence (4 categories)
Table S1.2	Baseline Characteristics of Study Population by Mediterranean Diet Adherence (tertiles)
Table S1.3	Baseline Characteristics of Study Population by Mediterranean Diet Adherence (quartiles)
Table S1.4	Baseline Characteristics of Study Population by Mediterranean Diet Adherence (3 categories) stratified by Sex
Table S1.5	Baseline Characteristics of Study Population and Older Adults Excluded from Analysis
Table S2.1	Modified Fried Frailty Phenotype by Mediterranean Diet adherence (3 category)
Table S2.2	Modified Fried Frailty Phenotype by Mediterranean Diet adherence (4 category)
Table S2.3	Modified Fried Frailty Phenotype by Mediterranean Diet adherence (tertiles)
Table S2.4	Modified Fried Frailty Phenotype by Mediterranean Diet adherence (quartiles)
Table S2.5	Prevalence of Modified Fried Frailty Phenotype Components
Table S2.6	Alternative Mediterranean Diet Food Groups using NHANES 24 hour diet recall
Table S3.1	Components of Mediterranean Diet by Modified Fried Frailty Phenotype Definitions (option 1)
Table S3.2	Prevalence of Frailty by Mediterranean Diet Adherence
Table S4.1	Univariate and Multivariate Logistic Regression of Frailty by Mediterranean Diet Score and Adherence (3 category)
Table S4.2	Univariate and Multivariate Logistic Regression of Frailty by Mediterranean Diet Score and Adherence (4 category)
Table S4.3	Univariate and Multivariate Logistic Regression of Frailty by Mediterranean Diet Score and Adherence (tertiles)
Table S4.4	Univariate and Multivariate Logistic Regression of Frailty by Mediterranean Diet Score and Adherence (quartiles)
Table S5.1	Univariate and Multivariate Logistic Regression of Frailty by Mediterranean Diet Score and Adherence – Sensitivity Analysis (3 category)
Table S5.2	Univariate and Multivariate Logistic Regression of Frailty by Mediterranean Diet Score and Adherence (4 category)
Table S5.3	Univariate and Multivariate Logistic Regression of Frailty by Mediterranean Diet Score and Adherence – Sensitivity Analysis (tertiles)
Table S5.4	Univariate and Multivariate Logistic Regression of Frailty by Mediterranean Diet Score and Adherence – Sensitivity Analysis (quartiles)

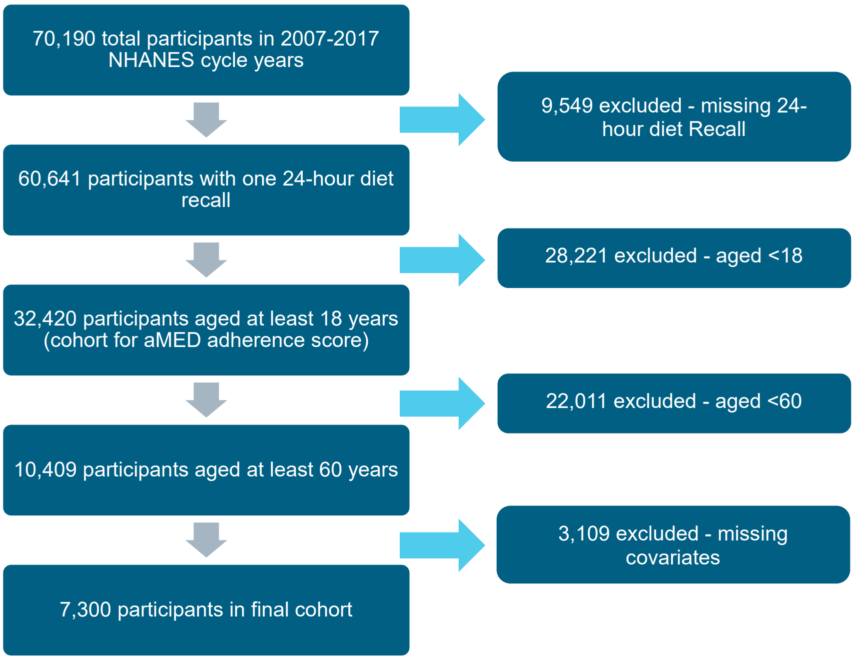

Figure S1: Participant Inclusion/Exclusion

Table S1: Baseline Characteristics of Study Population by Mediterranean Diet Adherence (3 categories) – with Confidence Intervals

Variable	Overall[1] N = 7,300 (100)	95%CI	Low[1] N = 2,434 (31)	95%CI	Moderate[1] N = 2,433 (33)	95%CI	High[1] N = 2,433 (36)	95%CI	p-value[2]
**Age (years)**	69.9 (6.7)	70, 70	69.4 (6.7)	69, 70	70.0 (6.8)	70, 70	70.1 (6.7)	69, 70	0.043
**Age Category (years)**									0.3
60-69	3,529 (51.5)	50, 53	1,189 (54.2)	50, 58	1,188 (50.6)	48, 53	1,152 (50.2)	48, 54	
70-79	2,451 (32.5)	31, 34	820 (30.6)	28, 35	811 (33.0)	31, 35	820 (33.7)	30, 36	
80-89	1,320 (15.9)	15, 17	425 (15.1)	13, 17	434 (16.4)	15, 18	461 (16.1)	14, 18	
**Sex (Female)**	3,697 (54.5)	53, 56	1,136 (51.6)	48, 54	1,270 (54.1)	52, 56	1,291 (57.3)	56, 61	0.016
**Race/Ethnicity**									<0.001
Non-Hispanic White	3,834 (80.0)	77, 82	1,328 (80.5)	77, 83	1,239 (79.7)	76, 82	1,267 (79.8)	78, 83	
Non-Hispanic Black	1,509 (8.1)	6.8, 9.6	544 (9.4)	7.7, 12	520 (8.5)	7.0, 10	445 (6.6)	5.3, 7.6	
Hispanic	1,449 (6.9)	5.6, 8.4	469 (7.0)	5.7, 9.2	538 (7.7)	6.2, 9.4	442 (6.1)	4.3, 6.8	
Other	508 (5.1)	4.2, 6.0	93 (3.1)	2.1, 3.8	136 (4.2)	3.6, 6.0	279 (7.5)	6.0, 9.4	
**BMI (kg/m2)**	29.6 (6.4)	29, 30	30.6 (7.0)	30, 31	29.7 (6.0)	29, 30	28.5 (6.0)	28, 29	<0.001
**BMI category (kg/m2)**									<0.001
<18.5	80 (0.9)	0.67, 1.2	27 (0.7)	0.48, 1.3	25 (1.0)	0.64, 1.4	28 (0.9)	0.52, 1.6	
18.5-24.9	1,579 (22.1)	21, 24	465 (18.4)	15, 21	479 (18.7)	17, 21	635 (28.3)	28, 34	
25-29.9	2,624 (35.8)	34, 37	860 (33.6)	30, 37	871 (37.7)	35, 39	893 (36.0)	33, 39	
30-34.9	1,788 (24.5)	23, 26	610 (25.8)	23, 30	614 (26.0)	24, 29	564 (22.1)	18, 23	
35-39.9	757 (10.5)	9.5, 12	288 (12.7)	10, 15	267 (10.6)	9.5, 13	202 (8.6)	6.7, 9.8	
>=40	472 (6.2)	5.3, 7.1	184 (8.7)	7.6, 12	177 (6.0)	4.9, 7.1	111 (4.2)	2.8, 5.0	
**Education Level**									<0.001
<=12th grade	1,964 (16.4)	15, 18	827 (22.9)	20, 25	685 (17.3)	15, 19	452 (10.0)	8.4, 12	
High school graduate/GED	1,792 (25.5)	24, 27	684 (30.4)	28, 34	609 (26.1)	24, 28	499 (20.8)	17, 23	
Some college or AA degree	1,997 (29.7)	28, 32	618 (29.4)	27, 34	681 (31.2)	28, 34	698 (28.5)	24, 31	
College graduate or above	1,547 (28.5)	26, 31	305 (17.3)	14, 21	458 (25.5)	22, 28	784 (40.7)	39, 47	
**Relationship Status[3]**	4,284 (65.2)	63, 67	1,351 (60.3)	56, 64	1,411 (66.3)	63, 68	1,522 (68.3)	66, 72	<0.001
**Income Poverty Level**	3.1 (1.6)	3.0, 3.2	2.8 (1.5)	2.6, 2.9	3.1 (1.6)	3.0, 3.2	3.4 (1.5)	3.4, 3.6	<0.001
**aMED Adherence Score**	3.6 (1.6)	3.6, 3.7	1.8 (0.8)	1.6, 1.6	3.5 (0.5)	3.5, 3.5	5.3 (0.9)	5.5, 5.6	<0.001
**Physically Active**	4,075 (62.5)	61, 64	1,174 (53.6)	50, 56	1,329 (59.3)	57, 63	1,572 (72.8)	72, 76	<0.001
**Smoking Status[4]**	3,822 (50.9)	49, 53	1,449 (58.3)	55, 62	1,232 (49.7)	48, 53	1,141 (45.8)	42, 49	<0.001
**Polypharmacy[5]**	3,210 (41.7)	40, 44	1,173 (46.6)	44, 50	1,117 (42.3)	41, 46	920 (36.9)	31, 38	<0.001
**Lives Alone**	1,906 (24.0)	23, 26	643 (25.9)	23, 29	634 (23.2)	22, 26	629 (23.1)	21, 25	0.2
**Medical Conditions[6]**	2.3 (1.4)	2.2, 2.3	2.4 (1.5)	2.4, 2.5	2.3 (1.4)	2.2, 2.4	2.1 (1.4)	2.0, 2.1	<0.001
Arthritis	3,980 (55.3)	54, 57	1,381 (58.2)	55, 63	1,312 (54.3)	52, 57	1,287 (53.7)	50, 57	0.10
Cancer	1,688 (26.4)	25, 28	566 (26.6)	24, 30	540 (25.9)	23, 28	582 (26.6)	24, 30	>0.9
Stroke	652 (7.7)	6.9, 8.7	256 (9.3)	7.9, 11	227 (8.0)	6.8, 9.3	169 (6.3)	4.7, 7.5	0.016
Pulmonary Disease	1,526 (21.8)	20, 23	558 (24.7)	23, 28	531 (22.9)	20, 25	437 (18.5)	15, 20	0.002
Cardiovascular Disease	1,295 (17.0)	16, 18	477 (17.5)	15, 20	425 (17.7)	16, 20	393 (15.8)	13, 18	0.4
Hypertension	5,033 (65.0)	63, 67	1,705 (65.8)	63, 69	1,713 (67.5)	64, 69	1,615 (61.9)	58, 65	0.023
Diabetes	2,036 (23.0)	22, 24	708 (24.3)	22, 27	704 (24.2)	23, 27	624 (20.6)	16, 21	0.035
Kidney Disease	479 (5.0)	4.4, 5.7	207 (7.3)	6.4, 9.8	156 (4.8)	3.7, 5.7	116 (3.2)	2.3, 4.1	<0.001
Depression	591 (6.9)	5.9, 7.9	249 (9.6)	8.1, 13	206 (7.1)	5.6, 8.2	136 (4.4)	2.9, 6.1	<0.001

1. Continuous aMED adherence score (range 0 to 9) used for aMED adherence categories: Low 1st tertile, Moderate 2nd tertile, and High 3rd tertile; descriptive statistics presented as unweighted N (weighted %); Mean (SD);
2. Chi-squared test with Rao & Scott's second-order correction; Kruskal-Wallis rank-sum test for complex survey samples;

Table S1: Baseline Characteristics of Study Population by Mediterranean Diet Adherence (3 categories) – with Confidence Intervals									
**Variable**	**Overall[1]** N = 7,300 (100)	**95%CI**	**Low[1]** N = 2,434 (31)	**95%CI**	**Moderate[1]** N = 2,433 (33)	**95%CI**	**High[1]** N = 2,433 (36)	**95%CI**	**p-value[2]**

3. Married or lives with partner (vs. widowed, separated, divorced, or single);
4. Current smoker or history of smoking 100 or more cigarettes in lifetime;
5. Taking 5 or more medications;
6. Summary score of comorbidities using self-reported history of arthritis, cancer, stroke, pulmonary disease, cardiovascular disease, hypertension, diabetes, kidney disease, and depression (range 0 to 9).

Abbreviations: AA (Associate of Arts), aMED (Alternative Mediterranean Diet); BMI (Body Mass Index), GED (General Education Degree), kg (kilogram), m (meter), N (number); SD (standard deviation); 95%CI (95% Confidence Interval)

Table S1.1: Baseline Characteristics of Study Population by Mediterranean Diet Adherence (4 categories)										
**Variable**	**Overall**[1] N = 7,300 (100)	**95%CI**	**Low**[1] N = 1,966 (26)	**95%CI**	**Low-Moderate**[1] N = 1,680 (23)	**95%CI**	**High-Moderate**[1] N = 1643 (22)	**95%CI**	**High**[1] N = 2,011 (30)	**95%CI**	**p-value**[2]
**Age (years)**	69.9 (6.7)	70, 70	69.3 (6.7)	69, 70	69.8 (6.8)	69, 70	70.5 (6.7)	70, 71	69.9 (6.7)	69, 70	0.011
**Age Category (years)**											0.2
60-69	3,529 (51.5)	50, 53	964 (53.7)	50, 58	850 (53.1)	50, 56	759 (48.0)	44, 52	956 (51.2)	48, 54	
70-79	2,451 (32.5)	31, 34	670 (31.7)	28, 35	524 (30.4)	27, 33	585 (35.5)	32, 39	672 (32.7)	30, 36	
80-89	1,320 (15.9)	15, 17	332 (14.6)	13, 17	306 (16.5)	15, 19	299 (16.6)	14, 19	383 (16.2)	14, 18	
**Sex (Female)**	3,697 (54.5)	53, 56	901 (50.9)	48, 54	856 (53.4)	50, 57	843 (54.2)	51, 58	1,097 (58.6)	56, 61	0.008
**Race/Ethnicity**											<0.001
Non-Hispanic White	3,834 (80.0)	77, 82	1,047 (80.5)	77, 83	840 (78.4)	75, 82	865 (80.0)	77, 83	1,082 (80.7)	78, 83	
Non-Hispanic Black	1,509 (8.1)	6.8, 9.6	451 (9.5)	7.7, 12	381 (9.2)	7.4, 11	319 (7.8)	6.3, 9.5	358 (6.3)	5.3, 7.6	
Hispanic	1,449 (6.9)	5.6, 8.4	387 (7.2)	5.7, 9.2	370 (7.7)	6.0, 9.8	349 (7.6)	6.0, 9.5	343 (5.4)	4.3, 6.8	
Other	508 (5.1)	4.2, 6.0	81 (2.8)	2.1, 3.8	89 (4.7)	3.3, 6.7	110 (4.6)	3.4, 6.2	228 (7.5)	6.0, 9.4	
**BMI (kg/m2)**	29.6 (6.4)	29, 30	30.8 (7.3)	30, 31	29.8 (5.7)	29, 30	29.7 (6.2)	29, 30	28.2 (5.9)	28, 29	<0.001
**BMI category (kg/m2)**											<0.001
<18.5	80 (0.9)	0.67, 1.2	23 (0.8)	0.48, 1.3	12 (0.6)	0.27, 1.4	24 (1.3)	0.83, 1.9	21 (0.9)	0.52, 1.6	
18.5-24.9	1,579 (22.1)	21, 24	364 (17.9)	15, 21	325 (18.1)	15, 21	336 (19.1)	17, 22	554 (30.8)	28, 34	
25-29.9	2,624 (35.8)	34, 37	689 (33.1)	30, 37	596 (36.8)	34, 40	600 (37.6)	35, 41	739 (36.0)	33, 39	
30-34.9	1,788 (24.5)	23, 26	499 (26.1)	23, 30	438 (26.9)	24, 30	400 (25.9)	23, 29	451 (20.4)	18, 23	
35-39.9	757 (10.5)	9.5, 12	234 (12.7)	10, 15	192 (11.7)	9.6, 14	167 (10.1)	8.4, 12	164 (8.1)	6.7, 9.8	
>=40	472 (6.2)	5.3, 7.1	157 (9.4)	7.6, 12	117 (5.8)	4.5, 7.5	116 (6.0)	4.6, 7.9	82 (3.8)	2.8, 5.0	
**Education Level**											<0.001
<=12th grade	1,964 (16.4)	15, 18	656 (22.2)	20, 25	513 (18.7)	16, 21	424 (15.9)	14, 19	371 (10.0)	8.4, 12	
High school graduate/GED	1,792 (25.5)	24, 27	559 (30.5)	28, 34	435 (27.7)	25, 31	397 (25.1)	22, 28	401 (19.9)	17, 23	
Some college or AA degree	1,997 (29.7)	28, 32	512 (30.1)	27, 34	457 (31.4)	28, 35	480 (30.8)	27, 34	548 (27.1)	24, 31	
College graduate or above	1,547 (28.5)	26, 31	239 (17.2)	14, 21	275 (22.2)	19, 26	342 (28.2)	25, 32	691 (43.1)	39, 47	
**Relationship Status**[3]	4,284 (65.2)	63, 67	1,074 (60.2)	56, 64	969 (65.4)	62, 69	973 (65.2)	62, 69	1,268 (69.3)	66, 72	0.001
**Income Poverty Level**	3.1 (1.6)	3.0, 3.2	2.8 (1.5)	2.6, 2.9	3.1 (1.5)	2.9, 3.2	3.1 (1.6)	3.0, 3.2	3.5 (1.5)	3.4, 3.6	<0.001
**aMED Adherence Score**	3.6 (1.6)	3.6, 3.7	1.6 (0.6)	1.6, 1.6	3.0 (0.0)	3.0, 3.0	4.0 (0.0)	4.0, 4.0	5.6 (0.8)	5.5, 5.6	
**Physically Active**	4,075 (62.5)	61, 64	946 (53.1)	50, 56	886 (59.7)	56, 63	924 (60.2)	57, 63	1,319 (74.1)	72, 76	<0.001
**Smoking Status**[4]	3,822 (50.9)	49, 53	1,191 (58.7)	55, 62	878 (49.8)	46, 53	826 (50.7)	47, 54	927 (45.2)	42, 49	<0.001
**Polypharmacy**[5]	3,210 (41.7)	40, 44	957 (46.8)	44, 50	779 (43.0)	39, 47	749 (44.4)	40, 48	725 (34.4)	31, 38	<0.001
**Lives Alone**	1,906 (24.0)	23, 26	518 (25.7)	23, 29	440 (23.6)	21, 26	428 (24.1)	21, 27	520 (22.8)	21, 25	0.4
**Medical Conditions**[6]	2.3 (1.4)	2.2, 2.3	2.5 (1.5)	2.4, 2.5	2.3 (1.4)	2.2, 2.5	2.3 (1.4)	2.2, 2.4	2.1 (1.4)	2.0, 2.1	<0.001
Arthritis	3,980 (55.3)	54, 57	1,125 (59.0)	55, 63	927 (55.7)	52, 60	862 (53.5)	50, 57	1,066 (53.2)	50, 57	0.087
Cancer	1,688 (26.4)	25, 28	469 (27.2)	24, 30	355 (24.4)	22, 27	380 (27.0)	23, 31	484 (26.8)	24, 30	0.6
Stroke	652 (7.7)	6.9, 8.7	216 (9.5)	7.9, 11	153 (7.6)	6.2, 9.4	152 (8.3)	6.6, 10	131 (6.0)	4.7, 7.5	0.013
Pulmonary Disease	1,526 (21.8)	20, 23	465 (25.5)	23, 28	367 (22.4)	19, 26	349 (22.5)	20, 26	345 (17.8)	15, 20	<0.001
Cardiovascular Disease	1,295 (17.0)	16, 18	407 (17.7)	15, 20	273 (16.7)	14, 20	305 (18.9)	16, 22	310 (15.2)	13, 18	0.2
Hypertension	5,033 (65.0)	63, 67	1,395 (65.9)	63, 69	1,179 (69.2)	66, 73	1,124 (64.1)	60, 68	1,335 (61.6)	58, 65	0.017
Diabetes	2,036 (23.0)	22, 24	580 (24.4)	22, 27	509 (24.6)	22, 28	455 (25.6)	22, 29	492 (18.5)	16, 21	<0.001
Kidney Disease	479 (5.0)	4.4, 5.7	185 (7.9)	6.4, 9.8	113 (5.1)	3.7, 6.9	92 (4.1)	3.2, 5.3	89 (3.1)	2.3, 4.1	<0.001
Depression	591 (6.9)	5.9, 7.9	218 (10.1)	8.1, 13	145 (8.4)	6.5, 11	121 (5.2)	3.9, 6.8	107 (4.2)	2.9, 6.1	<0.001

Table S1.1: Baseline Characteristics of Study Population by Mediterranean Diet Adherence (4 categories)										
**Variable**	**Overall**[1] N = 7,300 (100)	**95%CI**	**Low**[1] N = 1,966 (26)	**95%CI**	**Low-Moderate**[1] N = 1,680 (23)	**95%CI**	**High-Moderate**[1] N = 1643 (22)	**95%CI**	**High**[1] N = 2,011 (30)	**95%CI**	**p-value**[2]

1. Continuous aMED adherence score (range 0 to 9) used for aMED adherence categories: Low 0-2, Low-Moderate 3, High-Moderate 4, and High 5-9; descriptive statistics presented as unweighted N (weighted %); Mean (SD);
2. Chi-squared test with Rao & Scott's second-order correction; Kruskal-Wallis rank-sum test for complex survey samples;
3. Married or lives with partner (vs. widowed, separated, divorced, or single);
4. Current smoker or history of smoking 100 or more cigarettes in lifetime;
5. Taking 5 or more medications;
6. Summary score of comorbidities using self-reported history of arthritis, cancer, stroke, pulmonary disease, cardiovascular disease, hypertension, diabetes, kidney disease, and depression (range 0 to 9).

Abbreviations: AA (Associate of Arts), aMED (Alternative Mediterranean Diet); BMI (Body Mass Index), GED (General Education Degree), kg (kilogram), m (meter), N (number); SD (standard deviation); 95%CI (95% Confidence Interval)

Table S1.2: Baseline Characteristics of Study Population by Mediterranean Diet Adherence (tertiles)

Variable	Overall[1] N = 7,300 (100)	95%CI	Low[1] N = 2,434 (31)	95%CI	Moderate[1] N = 2,433 (33)	95%CI	High[1] N = 2,433 (36)	95%CI	p-value[2]
**Age (years)**	69.9 (6.7)	70, 70	69.4 (6.7)	69, 70	70.0 (6.8)	70, 70	70.1 (6.7)	70, 70	0.043
**Age Category (years)**									0.3
60-69	3,529 (51.5)	50, 53	1,189 (54.2)	51, 58	1,188 (50.6)	48, 54	1,152 (50.2)	47, 53	
70-79	2,451 (32.5)	31, 34	820 (30.6)	28, 34	811 (33.0)	30, 36	820 (33.7)	31, 36	
80-89	1,320 (15.9)	15, 17	425 (15.1)	13, 17	434 (16.4)	15, 18	461 (16.1)	14, 18	
**Sex (Female)**	3,697 (54.5)	53, 56	1,136 (51.6)	49, 54	1,270 (54.1)	52, 57	1,291 (57.3)	55, 60	0.016
**Race/Ethnicity**									<0.001
Non-Hispanic White	3,834 (80.0)	77, 82	1,328 (80.5)	77, 83	1,239 (79.7)	77, 82	1,267 (79.8)	77, 82	
Non-Hispanic Black	1,509 (8.1)	6.8, 9.6	544 (9.4)	7.7, 12	520 (8.5)	6.9, 10	445 (6.6)	5.5, 8.0	
Hispanic	1,449 (6.9)	5.6, 8.4	469 (7.0)	5.5, 8.9	538 (7.7)	6.1, 9.6	442 (6.1)	4.8, 7.6	
Other	508 (5.1)	4.2, 6.0	93 (3.1)	2.3, 4.2	136 (4.2)	3.1, 5.6	279 (7.5)	6.1, 9.3	
**BMI (kg/m2)**	29.6 (6.4)	29, 30	30.6 (7.0)	30, 31	29.7 (6.0)	29, 30	28.5 (6.0)	28, 29	<0.001
**BMI category (kg/m2)**									<0.001
<18.5	80 (0.9)	0.67, 1.2	27 (0.7)	0.47, 1.2	25 (1.0)	0.66, 1.6	28 (0.9)	0.54, 1.4	
18.5-24.9	1,579 (22.1)	21, 24	465 (18.4)	16, 21	479 (18.7)	16, 21	635 (28.3)	25, 31	
25-29.9	2,624 (35.8)	34, 37	860 (33.6)	30, 37	871 (37.7)	36, 40	893 (36.0)	33, 39	
30-34.9	1,788 (24.5)	23, 26	610 (25.8)	23, 29	614 (26.0)	23, 29	564 (22.1)	20, 24	
35-39.9	757 (10.5)	9.5, 12	288 (12.7)	11, 15	267 (10.6)	9.0, 12	202 (8.6)	7.2, 10	
>=40	472 (6.2)	5.3, 7.1	184 (8.7)	7.1, 11	177 (6.0)	4.9, 7.5	111 (4.2)	3.2, 5.4	
**Education Level**									<0.001
<=12th grade	1,964 (16.4)	15, 18	827 (22.9)	20, 26	685 (17.3)	15, 20	452 (10.0)	8.5, 12	
High school graduate/GED	1,792 (25.5)	24, 27	684 (30.4)	28, 33	609 (26.1)	24, 29	499 (20.8)	18, 23	
Some college or AA degree	1,997 (29.7)	28, 32	618 (29.4)	27, 32	681 (31.2)	28, 34	698 (28.5)	25, 32	
College graduate or above	1,547 (28.5)	26, 31	305 (17.3)	15, 20	458 (25.5)	23, 28	784 (40.7)	37, 45	
**Relationship Status[3]**	4,284 (65.2)	63, 67	1,351 (60.3)	57, 64	1,411 (66.3)	64, 69	1,522 (68.3)	65, 72	<0.001
**Income Poverty Level**	3.1 (1.6)	3.0, 3.2	2.8 (1.5)	2.7, 2.9	3.1 (1.6)	3.0, 3.2	3.4 (1.5)	3.3, 3.6	<0.001
**aMED Adherence Score**	3.6 (1.6)	3.6, 3.7	1.8 (0.8)	1.8, 1.9	3.5 (0.5)	3.4, 3.5	5.3 (0.9)	5.2, 5.4	<0.001
**Physically Active**	4,075 (62.5)	61, 64	1,174 (53.6)	51, 56	1,329 (59.3)	57, 62	1,572 (72.8)	70, 75	<0.001
**Smoking Status[4]**	3,822 (50.9)	49, 53	1,449 (58.3)	55, 61	1,232 (49.7)	47, 53	1,141 (45.8)	43, 49	<0.001
**Polypharmacy[5]**	3,210 (41.7)	40, 44	1,173 (46.6)	44, 50	1,117 (42.3)	39, 46	920 (36.9)	34, 40	<0.001
**Lives Alone**	1,906 (24.0)	23, 26	643 (25.9)	23, 29	634 (23.2)	21, 25	629 (23.1)	21, 26	0.2
**Medical Conditions[6]**	2.3 (1.4)	2.2, 2.3	2.4 (1.5)	2.3, 2.5	2.3 (1.4)	2.2, 2.4	2.1 (1.4)	2.0, 2.2	<0.001
Arthritis	3,980 (55.3)	54, 57	1,381 (58.2)	55, 61	1,312 (54.3)	51, 57	1,287 (53.7)	51, 57	0.10
Cancer	1,688 (26.4)	25, 28	566 (26.6)	24, 30	540 (25.9)	23, 29	582 (26.6)	24, 29	>0.9
Stroke	652 (7.7)	6.9, 8.7	256 (9.3)	7.7, 11	227 (8.0)	6.8, 9.4	169 (6.3)	5.0, 7.8	0.016
Pulmonary Disease	1,526 (21.8)	20, 23	558 (24.7)	22, 27	531 (22.9)	20, 26	437 (18.5)	16, 21	0.002
Cardiovascular Disease	1,295 (17.0)	16, 18	477 (17.5)	15, 20	425 (17.7)	16, 20	393 (15.8)	14, 18	0.4
Hypertension	5,033 (65.0)	63, 67	1,705 (65.8)	63, 68	1,713 (67.5)	64, 70	1,615 (61.9)	59, 65	0.023
Diabetes	2,036 (23.0)	22, 24	708 (24.3)	22, 27	704 (24.2)	22, 27	624 (20.6)	19, 23	0.035
Kidney Disease	479 (5.0)	4.4, 5.7	207 (7.3)	6.0, 8.9	156 (4.8)	3.8, 6.0	116 (3.2)	2.5, 4.2	<0.001
Depression	591 (6.9)	5.9, 7.9	249 (9.6)	7.9, 12	206 (7.1)	5.8, 8.6	136 (4.4)	3.2, 6.0	<0.001

Table S1.2: Baseline Characteristics of Study Population by Mediterranean Diet Adherence (tertiles)								
**Variable**	**Overall**[1] N = 7,300 (100)	**95%CI**	**Low**[1] N = 2,434 (31)	**95%CI**	**Moderate**[1] N = 2,433 (33)	**95%CI**	**High**[1] N = 2,433 (36)	**95%CI**	**p-value**[2]

1. Continuous aMED adherence score (range 0 to 9) used for aMED adherence categories: Low 1st tertile, Moderate 2nd tertile, and High 3rd tertile; descriptive statistics presented as unweighted N (weighted %); Mean (SD);
2. Chi-squared test with Rao & Scott's second-order correction; Kruskal-Wallis rank-sum test for complex survey samples;
3. Married or lives with partner (vs. widowed, separated, divorced, or single);
4. Current smoker or history of smoking 100 or more cigarettes in lifetime;
5. Taking 5 or more medications;
6. Summary score of comorbidities using self-reported history of arthritis, cancer, stroke, pulmonary disease, cardiovascular disease, hypertension, diabetes, kidney disease, and depression (range 0 to 9).

Abbreviations: AA (Associate of Arts), aMED (Alternative Mediterranean Diet); BMI (Body Mass Index), GED (General Education Degree), kg (kilogram), m (meter), N (number); SD (standard deviation); 95%CI (95% Confidence Interval)

Table S1.3: Baseline Characteristics of Study Population by Mediterranean Diet Adherence (quartiles)

Variable	Overall[1] N = 7,300 (100)	95%CI	Low[1] N = 1,825 (24)	95%CI	Low-Moderate[1] N = 1,825 (25)	95%CI	High-Moderate[1] N = 1,825 (24)	95%CI	High[1] N= 1,825 (28)	95%CI	p-value[2]
**Age (years)**	69.9 (6.7)	70, 70	69.3 (6.7)	69, 70	69.7 (6.7)	69, 70	70.4 (6.7)	70, 71	69.9 (6.7)	69, 70	0.010
**Age Category (years)**											0.4
60-69	3,529 (51.5)	50, 53	888 (54.1)	50, 58	929 (52.8)	49, 56	844 (48.6)	45, 52	868 (50.8)	47, 54	
70-79	2,451 (32.5)	31, 34	616 (30.7)	27, 34	579 (31.5)	29, 35	647 (34.9)	32, 38	609 (32.9)	30, 36	
80-89	1,320 (15.9)	15, 17	321 (15.2)	13, 17	317 (15.7)	14, 18	334 (16.5)	14, 19	348 (16.3)	14, 19	
**Sex (Female)**	3,697 (54.5)	53, 56	833 (50.8)	47, 54	927 (53.4)	50, 57	937 (54.3)	51, 57	1,000 (58.7)	56, 62	0.006
**Race/Ethnicity**											<0.001
Non-Hispanic White	3,834 (80.0)	77, 82	975 (80.7)	77, 84	914 (78.3)	75, 82	991 (80.7)	78, 83	954 (80.1)	77, 83	
Non-Hispanic Black	1,509 (8.1)	6.8, 9.6	418 (9.5)	7.6, 12	416 (9.2)	7.5, 11	342 (7.5)	6.1, 9.1	333 (6.5)	5.3, 7.8	
Hispanic	1,449 (6.9)	5.6, 8.4	363 (7.1)	5.6, 9.1	394 (7.7)	6.0, 9.8	377 (7.3)	5.8, 9.2	315 (5.5)	4.3, 7.0	
Other	508 (5.1)	4.2, 6.0	69 (2.7)	2.0, 3.7	101 (4.7)	3.4, 6.5	115 (4.5)	3.3, 5.9	223 (7.9)	6.3, 9.9	
**BMI (kg/m2)**	29.6 (6.4)	29, 30	30.8 (7.3)	30, 31	29.9 (5.8)	30, 30	29.5 (6.2)	29, 30	28.2 (5.9)	28, 29	<0.001
**BMI category (kg/m2)**											<0.001
<18.5	80 (0.9)	0.67, 1.2	22 (0.8)	0.50, 1.4	13 (0.6)	0.27, 1.3	27 (1.3)	0.88, 1.9	18 (0.8)	0.46, 1.6	
18.5-24.9	1,579 (22.1)	21, 24	342 (17.9)	15, 21	348 (18.1)	15, 21	387 (20.0)	18, 23	502 (30.9)	28, 34	
25-29.9	2,624 (35.8)	34, 37	645 (33.8)	30, 38	642 (35.9)	33, 39	668 (37.6)	35, 40	669 (35.9)	33, 39	
30-34.9	1,788 (24.5)	23, 26	460 (26.1)	23, 30	477 (26.8)	24, 30	439 (25.5)	23, 29	412 (20.4)	18, 23	
35-39.9	757 (10.5)	9.5, 12	208 (11.5)	9.3, 14	218 (12.9)	11, 15	183 (9.8)	8.1, 12	148 (8.3)	6.8, 10	
>=40	472 (6.2)	5.3, 7.1	148 (9.9)	8.0, 12	127 (5.7)	4.4, 7.2	121 (5.8)	4.5, 7.5	76 (3.8)	2.8, 5.1	
**Education Level**											<0.001
<=12th grade	1,964 (16.4)	15, 18	630 (23.1)	20, 26	539 (18.0)	16, 21	470 (16.1)	14, 19	325 (9.4)	7.7, 11	
High school graduate/GED	1,792 (25.5)	24, 27	508 (29.7)	27, 33	487 (28.7)	26, 32	441 (25.2)	23, 28	356 (19.4)	17, 23	
Some college or AA degree	1,997 (29.7)	28, 32	468 (30.5)	27, 34	503 (30.9)	27, 35	520 (30.2)	27, 34	506 (27.4)	24, 31	
College graduate or above	1,547 (28.5)	26, 31	219 (16.6)	14, 20	296 (22.4)	19, 26	394 (28.6)	25, 32	638 (43.8)	40, 48	
**Relationship Status**[3]	4,284 (65.2)	63, 67	1,008 (60.8)	57, 64	1,038 (64.4)	61, 68	1,092 (65.7)	62, 69	1,146 (69.2)	66, 73	0.004
**Income Poverty Level**	3.1 (1.6)	3.0, 3.2	2.7 (1.5)	2.6, 2.9	3.1 (1.6)	2.9, 3.2	3.1 (1.6)	3.0, 3.2	3.5 (1.5)	3.4, 3.6	<0.001
**aMED Adherence Score**	3.6 (1.6)	3.6, 3.7	1.6 (0.6)	1.5, 1.6	2.9 (0.3)	2.9, 2.9	4.1 (0.3)	4.1, 4.1	5.6 (0.8)	5.6, 5.7	<0.001
**Physically Active**	4,075 (62.5)	61, 64	868 (52.6)	49, 56	966 (59.7)	56, 63	1,043 (61.5)	58, 65	1,198 (74.1)	72, 76	<0.001
**Smoking Status**[4]	3,822 (50.9)	49, 53	1,107 (59.5)	56, 63	964 (49.8)	47, 53	909 (50.5)	47, 54	842 (45.0)	42, 48	<0.001
**Polypharmacy**[5]	3,210 (41.7)	40, 44	884 (47.0)	44, 50	854 (43.1)	39, 47	823 (44.2)	41, 48	649 (33.7)	30, 37	<0.001
**Lives Alone**	1,906 (24.0)	23, 26	476 (25.3)	23, 28	482 (24.1)	21, 27	474 (24.1)	21, 27	474 (22.7)	20, 25	0.6
**Medical Conditions**[6]	2.3 (1.4)	2.2, 2.3	2.4 (1.5)	2.4, 2.5	2.4 (1.4)	2.2, 2.5	2.3 (1.4)	2.2, 2.4	2.0 (1.4)	2.0, 2.1	<0.001
Arthritis	3,980 (55.3)	54, 57	1,036 (59.0)	55, 63	1,019 (55.9)	53, 59	965 (54.1)	51, 57	960 (52.6)	49, 56	0.071
Cancer	1,688 (26.4)	25, 28	439 (27.3)	24, 31	387 (24.5)	22, 27	423 (27.2)	24, 31	439 (26.4)	24, 29	0.6
Stroke	652 (7.7)	6.9, 8.7	198 (8.9)	7.3, 11	171 (8.4)	6.7, 10	166 (8.2)	6.7, 10	117 (5.8)	4.6, 7.4	0.037
Pulmonary Disease	1,526 (21.8)	20, 23	431 (25.2)	22, 28	401 (22.9)	20, 26	383 (22.3)	20, 25	311 (17.6)	15, 20	0.002
Cardiovascular Disease	1,295 (17.0)	16, 18	371 (17.5)	15, 20	309 (16.9)	14, 20	341 (18.7)	16, 21	274 (15.1)	13, 18	0.2
Hypertension	5,033 (65.0)	63, 67	1,293 (65.7)	63, 69	1,284 (69.2)	66, 73	1,236 (63.8)	60, 68	1,220 (61.6)	58, 65	0.019
Diabetes	2,036 (23.0)	22, 24	531 (24.2)	22, 27	560 (24.9)	22, 28	493 (24.9)	22, 28	452 (18.5)	16, 21	0.002

Table S1.3: Baseline Characteristics of Study Population by Mediterranean Diet Adherence (quartiles)

Variable	**Overall**[1] N = 7,300 (100)	95%CI	**Low**[1] N = 1,825 (24)	95%CI	**Low-Moderate**[1] N = 1,825 (25)	95%CI	**High-Moderate**[1] N = 1,825 (24)	95%CI	**High**[1] N= 1,825 (28)	95%CI	p-value[2]
Kidney Disease	479 (5.0)	4.4, 5.7	172 (7.9)	6.4, 9.7	126 (5.3)	4.0, 7.1	96 (4.0)	3.1, 5.1	85 (3.1)	2.3, 4.2	<0.001
Depression	591 (6.9)	5.9, 7.9	202 (9.8)	7.7, 12	161 (8.8)	7.0, 11	133 (5.1)	3.9, 6.6	95 (4.3)	2.9, 6.3	<0.001

1. Continuous aMED adherence score (range 0 to 9) used for aMED adherence categories: Low 1st quartile, Low-Moderate 2nd quartile, High-Moderate 3rd quartile, and High 4th quartile; descriptive statistics presented as unweighted N (weighted %); Mean (SD);
2. Chi-squared test with Rao & Scott's second-order correction; Kruskal-Wallis rank-sum test for complex survey samples;
3. Married or lives with partner (vs. widowed, separated, divorced, or single);
4. Current smoker or history of smoking 100 or more cigarettes in lifetime;
5. Taking 5 or more medications;
6. Summary score of comorbidities using self-reported history of arthritis, cancer, stroke, pulmonary disease, cardiovascular disease, hypertension, diabetes, kidney disease, and depression (range 0 to 9).

Abbreviations: AA (Associate of Arts), aMED (Alternative Mediterranean Diet); BMI (Body Mass Index), GED (General Education Degree), kg (kilogram), m (meter), N (number); SD (standard deviation); 95%CI (95% Confidence Interval)

Table S1.4: Baseline Characteristics of Study Population by Mediterranean Diet Adherence (3 categories) stratified by sex																		
Variable	Female									Male								
	Overall[1] N = 7,300 (100)	95%CI	Low[1] N = 2,434 (31)	95%CI	Moderate[1] N = 2,433 (33)	95%CI	High[1] N = 2,433 (36)	95%CI	p-value[2]	Overall[1] N =3,603 (100)	95%CI	Low[1] N = 2,434 (31)	95%CI	Moderate[1] N = 2,433 (33)	95%CI	High[1] N = 2,433 (36)	95%CI	p-value[2]
**Age (years)**	70.1 (6.8)	70, 70	69.5 (6.8)	69, 70	70.5 (6.8)	70, 71	69.9 (6.7)	69, 70	0.050	69.6 (6.6)	69, 70	69.2 (6.5)	69, 70	69.7 (6.6)	69, 70	69.9 (6.6)	69, 70	0.3
**Age Category (years)**									0.6									0.8
60-69	1,822 (50.3)	48, 53	449 (52.7)	46, 59	843 (48.7)	46, 52	530 (50.8)	47, 55		1,707 (53.0)	51, 55	515 (54.8)	50, 60	766 (52.7)	49, 57	426 (51.7)	47, 56	
70-79	1,200 (32.1)	30, 34	294 (31.0)	26, 36	540 (32.5)	30, 35	366 (32.3)	29, 36		1,251 (33.1)	31, 35	376 (32.4)	28, 38	569 (33.4)	30, 37	306 (33.2)	28, 38	
80-89	675 (17.6)	16, 19	158 (16.3)	13, 20	316 (18.8)	17, 21	201 (16.9)	15, 20		645 (13.9)	13, 15	174 (12.8)	11, 15	289 (13.9)	12, 16	182 (15.2)	13, 18	
**Race/Ethnicity**									<0.001									<0.001
Non-Hispanic White	1,905 (79.3)	77, 82	472 (79.8)	76, 83	845 (77.9)	75, 81	588 (80.9)	78, 83		1,929 (80.8)	78, 83	575 (81.2)	77, 85	860 (80.7)	77, 84	494 (80.5)	77, 83	
Non-Hispanic Black	775 (7.0)	5.7, 8.7	193 (7.5)	5.8, 9.8	388 (7.9)	6.3, 9.7	194 (5.6)	4.2, 7.3		674 (6.7)	5.4, 8.2	194 (7.0)	5.3, 9.1	331 (7.4)	5.8, 9.4	149 (5.3)	4.1, 6.8	
Hispanic	757 (8.7)	7.2, 10	198 (9.7)	7.9, 12	361 (9.5)	7.7, 12	198 (6.7)	5.4, 8.3		752 (7.4)	6.1, 9.0	253 (9.2)	7.0, 12	339 (7.3)	5.9, 9.1	160 (5.8)	4.6, 7.2	
Other	260 (5.0)	4.0, 6.2	38 (3.0)	1.9, 4.5	105 (4.8)	3.5, 6.5	117 (6.8)	5.3, 8.8		248 (5.1)	4.2, 6.3	43 (2.7)	2.0, 3.8	94 (4.6)	3.2, 6.5	111 (8.5)	6.4, 11	
**BMI (kg/m2)**	29.5 (6.9)	29, 30	31.0 (8.1)	30, 32	29.8 (6.4)	29, 30	28.1 (6.3)	28, 29	<0.001	29.6 (5.7)	29, 30	30.6 (6.3)	30, 31	29.7 (5.5)	29, 30	28.3 (5.2)	28, 29	<0.001
**BMI category (kg/m2)**									<0.001									<0.001
<18.5	47 (1.1)	0.80, 1.4	13 (0.9)	0.48, 1.8	20 (1.1)	0.73, 1.8	14 (1.1)	0.59, 2.0		33 (0.7)	0.40, 1.1	10 (0.7)	0.26, 1.7	16 (0.7)	0.34, 1.5	7 (0.6)	0.18, 2.2	
18.5-24.9	840 (25.2)	23, 27	161 (19.2)	16, 23	356 (21.9)	19, 25	323 (34.2)	30, 39		739 (18.3)	16, 20	203 (16.5)	13, 21	305 (14.8)	13, 17	231 (25.9)	22, 30	
25-29.9	1,175 (32.8)	31, 35	275 (33.0)	28, 38	542 (32.8)	30, 36	358 (32.7)	28, 37		1,449 (39.4)	37, 42	414 (33.3)	29, 38	654 (42.3)	39, 46	381 (40.7)	37, 45	
30-34.9	890 (22.5)	20, 25	228 (22.6)	19, 27	427 (25.6)	23, 29	235 (18.2)	15, 22		898 (27.0)	25, 30	271 (29.8)	25, 35	411 (27.4)	24, 31	216 (23.6)	20, 27	
35-39.9	445 (11.2)	9.8, 13	130 (13.6)	11, 17	204 (11.3)	9.4, 13	111 (9.2)	7.3, 12		312 (9.8)	8.4, 11	104 (11.7)	8.8, 15	155 (10.5)	8.5, 13	53 (6.6)	4.6, 9.4	
>=40	300 (7.3)	6.2, 8.5	94 (10.7)	7.8, 15	150 (7.4)	5.9, 9.1	56 (4.6)	3.2, 6.5		172 (4.9)	3.9, 6.0	63 (8.1)	5.8, 11	83 (4.3)	3.0, 6.0	26 (2.6)	1.4, 4.6	
**Education Level**									<0.001									<0.001
<=12th grade	1,009 (17.0)	15, 19	300 (22.0)	19, 26	493 (19.0)	17, 22	216 (10.6)	8.6, 13		955 (15.6)	14, 18	356 (22.5)	19, 26	444 (15.3)	13, 18	155 (9.1)	7.1, 11	
High school graduate/GED	948 (27.6)	26, 30	261 (32.7)	29, 37	444 (28.5)	26, 31	243 (22.7)	19, 26		844 (22.9)	21, 25	298 (28.2)	24, 33	388 (24.0)	21, 27	158 (15.8)	12, 20	
Some college or AA degree	1,079 (32.0)	29, 35	245 (30.9)	26, 36	504 (33.4)	30, 37	330 (30.8)	27, 35		918 (26.9)	25, 29	267 (29.3)	25, 34	433 (28.4)	25, 32	218 (21.9)	18, 26	
College graduate or above	661 (23.3)	21, 26	95 (14.5)	11, 18	258 (19.1)	16, 23	308 (35.8)	32, 40		886 (34.6)	31, 38	144 (20.0)	16, 25	359 (32.3)	28, 37	383 (53.3)	47, 59	
**Relationship Status[3]**	1,704 (54.6)	52, 57	386 (50.8)	46, 56	753 (52.5)	49, 56	565 (60.3)	56, 64	0.003	2,580 (77.9)	76, 80	688 (69.9)	66, 74	1,189 (80.3)	78, 83	703 (82.2)	78, 86	<0.001
**Income Poverty Level**	3.0 (1.6)	2.9, 3.1	2.7 (1.6)	2.5, 2.8	2.8 (1.5)	2.7, 3.0	3.4 (1.6)	3.2, 3.5	<0.001	3.3 (1.5)	3.2, 3.4	2.9 (1.5)	2.7, 3.0	3.3 (1.5)	3.2, 3.5	3.7 (1.4)	3.6, 3.9	<0.001
**aMED Adherence Score**	3.7 (1.6)	3.6, 3.8	1.6 (0.6)	1.5, 1.7	3.5 (0.5)	3.5, 3.5	5.6 (0.7)	5.5, 5.7	<0.001	3.5 (1.6)	3.4, 3.6	1.6 (0.6)	1.5, 1.6	3.5 (0.5)	3.5, 3.5	5.6 (0.8)	5.5, 5.7	<0.001
**Physically Active**	1,879 (57.7)	55, 60	398 (49.9)	46, 54	822 (53.7)	50, 57	659 (69.0)	65, 72	<0.001	2,196 (68.1)	66, 70	548 (56.5)	52, 61	988 (67.2)	64, 70	660 (81.4)	79, 84	<0.001
**Smoking Status[4]**	1,437 (40.2)	38, 43	419 (45.3)	40, 50	650 (41.1)	38, 44	368 (35.2)	31, 40	0.007	2,385 (63.7)	61, 66	772 (72.6)	69, 76	1,054 (60.9)	57, 65	559 (59.3)	55, 64	<0.001
**Polypharmacy[5]**	1,612 (41.7)	40, 44	455 (48.5)	44, 53	757 (42.6)	39, 46	400 (35.4)	31, 40	<0.001	1,598 (41.6)	39, 44	502 (45.0)	40, 50	771 (44.8)	41, 49	325 (32.9)	29, 37	<0.001
**Lives Alone**	1,175 (30.3)	28, 32	270 (30.1)	26, 35	541 (31.0)	28, 34	364 (29.7)	27, 33	0.8	731 (16.4)	15, 18	248 (21.2)	18, 25	327 (15.5)	13, 18	156 (13.1)	10, 17	0.002
**Medical Conditions[6]**	2.3 (1.4)	2.2, 2.4	2.5 (1.5)	2.4, 2.6	2.4 (1.4)	2.3, 2.5	2.0 (1.4)	1.9, 2.1	<0.001	2.3 (1.4)	2.2, 2.3	2.4 (1.5)	2.3, 2.6	2.3 (1.4)	2.1, 2.4	2.1 (1.3)	2.0, 2.2	0.040
Arthritis	2,298 (61.3)	59, 63	603 (65.3)	61, 70	1,052 (61.7)	58, 65	643 (57.8)	53, 62	0.067	1,682 (48.1)	45, 51	522 (52.3)	47, 57	737 (46.4)	42, 50	423 (46.6)	42, 52	0.14
Cancer	778 (24.5)	23, 26	200 (24.6)	21, 29	342 (24.9)	22, 28	236 (23.9)	21, 27	0.9	910 (28.6)	26, 31	269 (29.8)	25, 35	393 (26.5)	23, 31	248 (30.9)	27, 35	0.3
Stroke	308 (7.7)	6.6, 9.0	90 (8.8)	6.9, 11	152 (8.6)	6.9, 11	66 (5.7)	4.2, 7.6	0.029	344 (7.8)	6.5, 9.3	126 (10.2)	7.6, 13	153 (7.1)	5.6, 9.1	65 (6.3)	4.5, 8.9	0.043
Pulmonary Disease	863 (24.9)	23, 27	258 (30.6)	26, 35	402 (26.5)	23, 30	203 (18.6)	16, 22	<0.001	663 (18.1)	16, 20	207 (20.2)	17, 24	314 (17.8)	15, 21	142 (16.6)	13, 21	0.4
Cardiovascular Disease	482 (12.2)	11, 14	150 (13.4)	11, 16	224 (13.7)	11, 16	108 (9.5)	7.1, 12	0.035	813 (22.7)	21, 25	257 (22.3)	19, 26	354 (22.5)	20, 25	202 (23.5)	20, 28	0.9
Hypertension	2,621 (66.1)	64, 69	666 (68.5)	65, 72	1,208 (67.7)	64, 71	747 (62.2)	58, 66	0.041	2,412 (63.6)	61, 66	729 (63.1)	58, 68	1,095 (65.5)	62, 69	588 (60.7)	56, 65	0.4
Diabetes	925 (19.5)	18, 21	254 (21.2)	18, 25	431 (21.1)	18, 24	240 (15.9)	13, 19	0.014	1,111 (27.2)	25, 29	326 (27.8)	24, 32	533 (29.8)	27, 33	252 (22.3)	19, 26	0.018
Kidney Disease	222 (4.7)	4.0, 5.7	77 (7.2)	5.3, 9.7	88 (4.2)	2.9, 5.9	57 (3.7)	2.6, 5.3	0.016	257 (5.3)	4.6, 6.2	108 (8.7)	6.7, 11	117 (5.1)	4.0, 6.5	32 (2.2)	1.4, 3.4	<0.001
Depression	363 (8.1)	7.0, 9.5	123 (11.9)	9.3, 15	167 (8.0)	6.6, 9.8	73 (5.5)	3.7, 8.2	0.002	228 (5.4)	4.3, 6.7	95 (8.4)	5.8, 12	99 (5.3)	3.7, 7.5	34 (2.4)	1.5, 3.9	0.001

1. Continuous aMED adherence score (range 0 to 9) used for aMED adherence categories: Low 1st tertile, Moderate 2nd tertile, and High 3rd tertile; descriptive statistics presented as unweighted N (weighted %); Mean (SD);
2. Chi-squared test with Rao & Scott's second-order correction; Kruskal-Wallis rank-sum test for complex survey samples;
3. Married or lives with partner (vs. widowed, separated, divorced, or single);
4. Current smoker or history of smoking 100 or more cigarettes in lifetime;
5. Taking 5 or more medications;
6. Summary score of comorbidities using self-reported history of arthritis, cancer, stroke, pulmonary disease, cardiovascular disease, hypertension, diabetes, kidney disease, and depression (range 0 to 9).

Abbreviations: AA (Associate of Arts), aMED (Alternative Mediterranean Diet); BMI (Body Mass Index), GED (General Education Degree), kg (kilogram), m (meter), N (number); SD (standard deviation); 95%CI (95% Confidence Interval)

Table S1.5: Baseline Characteristics of Study Population and Older Adults Excluded from Analysis							
**Variable**	**Overall[1]** N = 11,910 (100)	**95%CI**	**Excluded[1]** N = 4,610 (34)	**95%CI**	**Included[1]** N = 7,300 (66)	**95%CI**	**p-value[2]**
**Age (years)**	69.8 (6.9)	70, 70	70.1 (7.2)	70, 71	69.6 (6.7)	69, 70	0.013
**Age Category (years)**							0.2
60-69	5,861 (52.5)	51, 54	2,332 (51.3)	49, 54	3,529 (53.2)	51, 55	
70-79	3,667 (30.2)	29, 31	1,216 (26.6)	25, 29	2,451 (32.0)	31, 33	
80-89	2,382 (17.3)	16, 18	1,062 (22.1)	20, 24	1,320 (14.9)	14, 16	
**Sex (Female)**	6,065 (55.0)	54, 56	2,368 (56.4)	54, 58	3,697 (54.3)	53, 56	0.14
**Race/Ethnicity**							<0.001
Non-Hispanic White	5,608 (76.7)	74, 79	1,774 (69.0)	66, 72	3,834 (80.6)	78, 83	
Non-Hispanic Black	2,543 (9.0)	6.6, 9.5	1,234 (10.6)	9.0, 13	1,449 (6.6)	5.3, 8.1	
Hispanic	2,683 (8.0)	7.7, 10	1,034 (11.0)	9.5, 13	1,509 (8.0)	6.8, 9.5	
Other	1,076 (6.3)	5.4, 7.3	568 (9.4)	7.9, 11	508 (4.8)	4.0, 5.7	
**BMI (kg/m2)**	29.2 (6.3)	29, 29	28.2 (5.9)	28, 29	29.6 (6.4)	29, 30	<0.001
**BMI category (kg/m2)**							<0.001
<18.5	150 (1.2)	0.93, 1.4	70 (1.7)	1.2, 2.4	80 (0.9)	0.71, 1.2	
18.5-24.9	2,668 (24.0)	23, 25	1,089 (29.0)	27, 31	1,579 (21.9)	20, 23	
25-29.9	3,993 (35.8)	35, 37	1,369 (36.8)	35, 39	2,624 (35.4)	34, 37	
30-34.9	2,553 (23.2)	22, 25	765 (19.8)	18, 22	1,788 (24.6)	23, 26	
35-39.9	1,073 (10.1)	9.3, 11	316 (8.3)	7.0, 9.8	757 (10.9)	9.8, 12	
>=40	643 (5.7)	5.1, 6.4	171 (4.4)	3.5, 5.4	472 (6.3)	5.5, 7.2	
**Education Level**							<0.001
<=12th grade	3,685 (18.6)	17, 20	1,721 (23.8)	22, 26	1,964 (16.1)	15, 18	
High school graduate/GED	2,774 (24.9)	23, 26	982 (22.9)	21, 25	1,792 (25.9)	24, 28	
Some college or AA degree	3,031 (28.5)	27, 30	1,034 (26.6)	25, 29	1,997 (29.5)	28, 31	
College graduate or above	2,387 (28.0)	26, 30	840 (26.8)	24, 29	1,547 (28.6)	26, 31	
**Relationship Status[3]**	6,723 (62.8%)	61, 64	2,439 (57.9)	56, 60	4,284 (65.3)	63, 67	<0.001
**Income Poverty Level**	3.1 (1.6)	3.0, 3.1	2.8 (1.6)	2.7, 2.9	3.1 (1.6)	3.0, 3.2	<0.001
**Physically Active**	6,195 (59.5)	58, 61	2,120 (52.9)	51, 55	4,075 (62.8)	61, 65	<0.001
**Smoking Status[4]**	5,985 (50.1)	49, 52	2,163 (47.4)	45, 50	3,822 (51.5)	50, 53	0.006
**Polypharmacy[5]**	4,491 (42.6)	41, 44	1,281 (45.6)	43, 49	3,210 (41.7)	40, 44	0.012
**Lives Alone**	2,990 (23.8)	23, 25	1,084 (24.0)	22, 26	1,906 (23.7)	22, 25	0.8
**Medical Conditions[6]**	2.1 (1.5)	2.1, 2.1	1.8 (1.5)	1.7, 1.8	2.3 (1.4)	2.2, 2.3	<0.001
Arthritis	5,952 (51.9)	51, 53	1,972 (45.4)	43, 47	3,980 (55.3)	54, 57	<0.001
Cancer	2,451 (24.9)	24, 26	763 (21.4)	20, 23	1,688 (26.6)	25, 28	<0.001
Stroke	1,034 (7.6)	6.9, 8.3	382 (7.7)	6.7, 8.8	652 (7.5)	6.7, 8.4	0.8
Pulmonary Disease	2,232 (20.1)	19, 21	706 (16.6)	15, 19	1,526 (21.9)	20, 23	<0.001
Cardiovascular Disease	1,882 (15.7)	15, 17	587 (12.9)	12, 14	1,295 (17.1)	16, 18	<0.001
Hypertension	7,325 (58.6)	57, 60	2,292 (46.1)	44, 48	5,033 (64.9)	63, 67	<0.001
Diabetes	2,965 (20.6)	20, 22	929 (16.4)	15, 18	2,036 (22.7)	21, 24	<0.001
Kidney Disease	738 (5.1)	4.5, 5.8	259 (5.0)	4.2, 5.9	479 (5.2)	4.6, 5.9	0.7
Depression	847 (6.9)	6.1, 7.7	256 (6.5)	5.4, 7.8	591 (7.0)	6.0, 8.1	0.5

1. Adults 60 years and older; excluded for missing diet recall data; descriptive statistics presented as unweighted N (weighted %); Mean (SD);
2. Chi-squared test with Rao & Scott's second-order correction; Kruskal-Wallis rank-sum test for complex survey samples;
3. Married or lives with partner (vs. widowed, separated, divorced, or single);
4. Current smoker or history of smoking 100 or more cigarettes in lifetime;
5. Taking 5 or more medications;
6. Summary score of comorbidities using self-reported history of arthritis, cancer, stroke, pulmonary disease, cardiovascular disease, hypertension, diabetes, kidney disease, and depression (range 0 to 9).

Abbreviations: AA (Associate of Arts), aMED (Alternative Mediterranean Diet); BMI (Body Mass Index), GED (General Education Degree), kg (kilogram), m (meter), N (number); SD (standard deviation); 95%CI (95% Confidence Interval)

Table S2.1: Components of Modified Fried Frailty Phenotype by Mediterranean Diet adherence (3 category)					
	**Overall**[1] N = 7,300 (100)	**Low**[1] N = 1,966 (26)	**Moderate**[1] N = 3,323 (45)	**High**[1] N = 2,011 (30)	**p-value**[2]
**Frailty Indicators**[3]					
Low Physical Activity	1,368 (21.1)	381 (22.3)	625 (21.3)	362 (19.7)	0.4
Exhaustion	1,330 (16.9)	433 (21.8)	605 (16.6)	292 (13.1)	<0.001
Slow Walking Speed	780 (8.5)	279 (12.3)	340 (7.9)	161 (6.1)	<0.001
Weakness	1,993 (22.9)	617 (27.6)	921 (22.9)	455 (18.8)	<0.001
Unintentional Weight Loss	693 (8.0)	254 (10.0)	293 (7.7)	146 (6.7)	0.034
Weight loss	1,630 (21.0)	490 (21.8)	729 (21.6)	411 (19.4)	0.4
Low BMI	80 (0.9)	23 (0.8)	36 (0.9)	21 (0.9)	0.9
**Definition 1**					<0.001
Robust	3,517 (51.0)	839 (45.1)	1,592 (50.8)	1,086 (56.3)	
Pre-frail	3,173 (41.9)	907 (44.3)	1,455 (42.2)	811 (39.4)	
Frail	610 (7.1)	220 (10.6)	276 (7.0)	114 (4.3)	
**Definition 2**					<0.001
Robust	3,024 (44.3)	723 (38.8)	1,359 (43.6)	942 (50.0)	
Pre-frail	3,537 (46.9)	990 (49.2)	1,629 (47.8)	918 (43.7)	
Frail	739 (8.8)	253 (12.0)	335 (8.6)	151 (6.3)	
**Definition 3**					<0.001
Robust	3,748 (53.6)	915 (48.0)	1,699 (53.4)	1,134 (58.9)	
Pre-frail	3,063 (40.5)	882 (43.3)	1,396 (40.9)	785 (37.7)	
Frail	489 (5.8)	169 (8.7)	228 (5.8)	92 (3.4)	

1. Continuous aMED adherence score (range 0 to 9) used for aMED adherence categories: Low 0-2, Moderate 3-4, and High 5-9; descriptive statistics presented as unweighted N (weighted %);
2. Chi-squared test with Rao & Scott's second-order correction;
3. Modified Fried Frailty Phenotype is based on 5 indicators (a) self-reported weakness: difficulty with lifting or carrying something as heavy as 10 pounds; (b) self-reported low physical activity: top quintile of minutes of sedentary activity; (c) self-reported exhaustion: feelings of tiredness or having little energy over the past two weeks for "more than half the days" or "nearly every day"; (d) self-reported slow walking speed: difficulty walking between rooms on the same floor; and (e) self-reported "weight loss" evaluated in three ways (i) Definition 1: unintentional weight loss ≥ 10lbs in the previous year; (ii) Definition 2: any weight loss ≥ 10lbs in the previous year; and (iii) Definition 3: low BMI defined as ≤18.5 kg/m^2;

Abbreviations: aMED (alternative Mediterranean Diet); BMI (body mass index); kg (kilogram); lb (pound); N (number)

Table S2.2: Components of Modified Fried Frailty Phenotype by Mediterranean Diet adherence (4 category)						
	**Overall**[1] N = 7,300 (100)	**Low**[1] N = 1,966 (26)	**Low-Moderate**[1] N = 1,680 (23)	**High-Moderate**[1] N = 1,643 (22)	**High**[1] N = 2,011 (30)	**p-value**[2]
**Frailty Indicators**[3]						
Low Physical Activity	1,368 (21.1)	381 (22.3)	316 (21.3)	309 (21.3)	362 (19.7)	0.6
Exhaustion	1,330 (16.9)	433 (21.8)	320 (18.3)	285 (14.9)	292 (13.1)	<0.001
Slow Walking Speed	780 (8.5)	279 (12.3)	182 (8.4)	158 (7.3)	161 (6.1)	<0.001
Weakness	1,993 (22.9)	617 (27.6)	480 (23.9)	441 (22.0)	455 (18.8)	<0.001
Unintentional Weight Loss	693 (8.0)	254 (10.0)	157 (8.5)	136 (6.8)	146 (6.7)	0.029
Weight loss	1,630 (21.0)	490 (21.8)	372 (21.7)	357 (21.5)	411 (19.4)	0.5
Low BMI	80 (0.9)	23 (0.8)	12 (0.6)	24 (1.3)	21 (0.9)	0.4
**Definition 1**						<0.001
Robust	3,517 (51.0)	839 (45.1)	806 (50.0)	786 (51.6)	1,086 (56.3)	
Pre-frail	3,173 (41.9)	907 (44.3)	711 (41.5)	744 (42.8)	811 (39.4)	
Frail	610 (7.1)	220 (10.6)	163 (8.5)	113 (5.6)	114 (4.3)	
**Definition 2**						<0.001
Robust	3,024 (44.3)	723 (38.8)	687 (42.9)	672 (44.3)	942 (50.0)	
Pre-frail	3,537 (46.9)	990 (49.2)	805 (47.6)	824 (48.1)	918 (43.7)	
Frail	739 (8.8)	253 (12.0)	188 (9.5)	147 (7.6)	151 (6.3)	
**Definition 3**						<0.001
Robust	3,748 (53.6)	915 (48.0)	865 (52.7)	834 (54.1)	1,134 (58.9)	
Pre-frail	3,063 (40.5)	882 (43.3)	679 (40.5)	717 (41.2)	785 (37.7)	
Frail	489 (5.8)	169 (8.7)	136 (6.8)	92 (4.7)	92 (3.4)	

1. Continuous aMED adherence score (range 0 to 9) used for aMED adherence categories: Low 0-2, Low-Moderate 3, High-Moderate 4, and High 5-9; descriptive statistics presented as unweighted N (weighted %);
2. Chi-squared test with Rao & Scott's second-order correction;
3. Modified Fried Frailty Phenotype is based on 5 indicators (a) self-reported weakness: difficulty with lifting or carrying something as heavy as 10 pounds; (b) self-reported low physical activity: top quintile of minutes of sedentary activity; (c) self-reported exhaustion: feelings of tiredness or having little energy over the past two weeks for “more than half the days” or “nearly every day”; (d) self-reported slow walking speed: difficulty walking between rooms on the same floor; and (e) self-reported “weight loss” evaluated in three ways (i) Definition 1: unintentional weight loss ≥ 10lbs in the previous year; (ii) Definition 2: any weight loss ≥ 10lbs in the previous year; and (iii) Definition 3: low BMI defined as ≤18.5 kg/m^2;

Abbreviations: aMED (alternative Mediterranean Diet); BMI (body mass index); kg (kilogram); lb (pound); N (number)

Table S2.3: Components of Modified Fried Frailty Phenotype by Mediterranean Diet adherence (tertiles)					
	**Overall**[1] N = 7300 (100)	**Low**[1] N = 2434 (31)	**Moderate**[1] N = 2433 (33)	**High**[1] N = 2433 (36)	**p-value**[2]
**Frailty Indicators**[3]					
Low Physical Activity	1,368 (21.1)	455 (21.6)	480 (22.3)	433 (19.5)	0.2
Exhaustion	1,330 (16.9)	505 (20.7)	466 (17.1)	359 (13.3)	<0.001
Slow Walking Speed	780 (8.5)	323 (11.5)	255 (8.0)	202 (6.3)	<0.001
Weakness	1,993 (22.9)	738 (26.3)	670 (23.0)	585 (20.0)	0.003
Unintentional Weight Loss	693 (8.0)	286 (9.3)	223 (8.1)	184 (6.7)	0.079
Weight loss	1,630 (21.0)	580 (21.2)	537 (21.5)	513 (20.4)	0.8
Low BMI	80 (0.9)	27 (0.7)	25 (1.0)	28 (0.9)	0.6
**Definition 1**					<0.001
Robust	3,517 (51.0)	1,091 (47.0)	1,141 (50.0)	1,285 (55.3)	
Pre-frail	3,173 (41.9)	1,086 (43.1)	1,080 (42.4)	1,007 (40.4)	
Frail	610 (7.1)	257 (9.9)	212 (7.6)	141 (4.3)	
**Definition 2**					<0.001
Robust	3,024 (44.3)	942 (40.6)	980 (43.0)	1,102 (48.6)	
Pre-frail	3,537 (46.9)	1,196 (48.3)	1,194 (47.9)	1,147 (45.0)	
Frail	739 (8.8)	296 (11.2)	259 (9.1)	184 (6.5)	
**Definition 3**					<0.001
Robust	3,748 (53.6)	1,178 (49.7)	1,228 (52.7)	1,342 (57.9)	
Pre-frail	3,063 (40.5)	1,057 (42.2)	1,028 (41.0)	978 (38.7)	
Frail	489 (5.8)	199 (8.1)	177 (6.3)	113 (3.4)	

1. Continuous aMED adherence score (range 0 to 9) used for aMED adherence categories: Low 1st tertile, Moderate 2nd tertile, and High 3rd tertile; descriptive statistics presented as unweighted N (weighted %);
2. Chi-squared test with Rao & Scott's second-order correction;
3. Modified Fried Frailty Phenotype is based on 5 indicators (a) self-reported weakness: difficulty with lifting or carrying something as heavy as 10 pounds; (b) self-reported low physical activity: top quintile of minutes of sedentary activity; (c) self-reported exhaustion: feelings of tiredness or having little energy over the past two weeks for “more than half the days” or “nearly every day”; (d) self-reported slow walking speed: difficulty walking between rooms on the same floor; and (e) self-reported “weight loss” evaluated in three ways (i) Definition 1: unintentional weight loss ≥ 10lbs in the previous year; (ii) Definition 2: any weight loss ≥ 10lbs in the previous year; and (iii) Definition 3: low BMI defined as ≤18.5 kg/m^2;

Abbreviations: aMED (alternative Mediterranean Diet); BMI (body mass index); kg (kilogram); lb (pound); N (number)

Table S2.4: Components of Modified Fried Frailty Phenotype by Mediterranean Diet adherence (quartiles)						
	**Overall**[1] N = 7,300 (100)	**Low**[1] N = 1,825 (24)	**Low-Moderate**[1] N = 1,825 (25)	**High-Moderate**[1] N = 1,825 (24)	**High**[1] N = 1,825 (28)	**p-value**[2]
**Frailty Indicators**[3]						
Low Physical Activity	1,368 (21.1)	357 (22.2)	340 (21.4)	337 (21.1)	334 (19.7)	0.6
Exhaustion	1,330 (16.9)	401 (21.6)	353 (18.8)	325 (15.1)	251 (12.7)	<0.001
Slow Walking Speed	780 (8.5)	256 (12.3)	206 (8.7)	170 (7.2)	148 (6.1)	<0.001
Weakness	1,993 (22.9)	563 (27.6)	534 (24.2)	489 (22.0)	407 (18.6)	<0.001
Unintentional Weight Loss	693 (8.0)	237 (10.1)	174 (8.4)	153 (7.0)	129 (6.6)	0.031
Weight loss	1,630 (21.0)	454 (21.8)	408 (21.7)	388 (21.1)	380 (19.7)	0.7
Low BMI	80 (0.9)	22 (0.8)	13 (0.6)	27 (1.3)	18 (0.8)	0.3
**Definition 1**						<0.001
Robust	3,517 (51.0)	786 (44.9)	861 (49.7)	882 (52.1)	988 (56.3)	
Pre-frail	3,173 (41.9)	833 (44.6)	787 (41.5)	816 (42.1)	737 (39.7)	
Frail	610 (7.1)	206 (10.5)	177 (8.8)	127 (5.7)	100 (4.1)	
**Definition 2**						<0.001
Robust	3,024 (44.3)	678 (38.7)	734 (42.6)	760 (45.0)	852 (49.8)	
Pre-frail	3,537 (46.9)	913 (49.5)	884 (47.5)	904 (47.3)	836 (44.0)	
Frail	739 (8.8)	234 (11.8)	207 (9.9)	161 (7.6)	137 (6.3)	
**Definition 3**						<0.001
Robust	3,748 (53.6)	856 (47.8)	926 (52.4)	934 (54.5)	1,032 (59.0)	
Pre-frail	3,063 (40.5)	811 (43.7)	752 (40.4)	789 (40.8)	711 (37.8)	
Frail	489 (5.8)	158 (8.5)	147 (7.1)	102 (4.7)	82 (3.3)	

1. Continuous aMED adherence score (range 0 to 9) used for aMED adherence categories: Low 1st quartile, Low-Moderate 2nd quartile, High-Moderate 3rd quartile, and High 4th quartile; descriptive statistics presented as unweighted N (weighted %);
2. Chi-squared test with Rao & Scott's second-order correction;
3. Modified Fried Frailty Phenotype is based on 5 indicators (a) self-reported weakness: difficulty with lifting or carrying something as heavy as 10 pounds; (b) self-reported low physical activity: top quintile of minutes of sedentary activity; (c) self-reported exhaustion: feelings of tiredness or having little energy over the past two weeks for “more than half the days” or “nearly every day”; (d) self-reported slow walking speed: difficulty walking between rooms on the same floor; and (e) self-reported “weight loss” evaluated in three ways (i) Definition 1: unintentional weight loss ≥ 10lbs in the previous year; (ii) Definition 2: any weight loss ≥ 10lbs in the previous year; and (iii) Definition 3: low BMI defined as ≤18.5 kg/m^2;

Abbreviations: aMED (alternative Mediterranean Diet); BMI (body mass index); kg (kilogram); lb (pound); N (number)

Table S2.5: Prevalence of Frailty By Indicators				
**Frailty Indicators[1]**	**Definition 1**	**Definition 2**	**Definition 3**	**p-value[2]**
Weight loss	693 (8.0)	1,630 (21.0)	80 (0.9)	<0.001
Weakness	1,993 (22.9)	1,993 (22.9)	1,993 (22.9)	--
Low physical activity	1,368 (21.1)	1,368 (21.1)	1,368 (21.1)	--
Exhaustion	1,330 (16.9)	1,330 (16.9)	1,330 (16.9)	--
Slow walking speed	780 (8.5)	780 (8.5)	780 (8.5)	--
**Number of indicators[3]**				
0	3,517 (48.2)	3,024 (41.4)	3,748 (51.3)	<0.001
1	2,172 (29.8)	2,407 (33.0)	2,132 (29.2)	<0.001
2	1,001 (13.7)	1,130 (15.5)	931 (12.8)	<0.001
3	460 (6.3)	547 (7.5)	399 (5.5)	<0.001
4	140 (1.9)	167 (2.3)	90 (1.2)	<0.001
5	10 (0.1)	25 (0.3)	0 (0)	<0.001
**Frailty Status[4]**				
Robust	3,517 (51.0)	3,024 (44.3)	3,748 (53.6)	<0.001
Pre-frail	3,173 (41.9)	3,537 (46.9)	3,063 (40.5)	<0.001
Frail	610 (7.1)	739 (8.8)	489 (5.8)	<0.001

1. Frailty status is based on 5 indicators (a) self-reported weakness: difficulty with lifting or carrying something as heavy as 10 pounds; (b) self-reported low physical activity: top quintile of minutes of sedentary activity; (c) self-reported exhaustion: feelings of tiredness or having little energy over the past two weeks for “more than half the days” or “nearly every day”; (d) self-reported slow walking speed: difficulty walking between rooms on the same floor; and (e) self-reported “weight loss” evaluated in three ways (i) Definition 1: unintentional weight loss ≥ 10lbs in the previous year; (ii) Definition 2: any weight loss ≥ 10lbs in the previous year; and (iii) Definition 3: low BMI defined as ≤18.5 kg/m^2; total N = 7,300; descriptive statistics presented as unweighted N (unweighted %); 2. Chi-square test of independence;
3. descriptive statistics for each definitions presented as unweighted N (unweighted %);
4. Robust: 0 indicators; Pre-frail: 1 to 2 indicators; Frail: 3 or more indicators;

Abbreviations: aMED (Alternative Mediterranean Diet); BMI (Body Mass Index), kg (kilogram), lb (pound), m (meter), N (number)

Table S2.6: Mediterranean Diet Adherence Food Groups using NHANES 24-hour Diet Recall		
**Component**	**Description**	**Scoring Criteria**
**Fruit**	Total intact fruits (whole or cut) and fruit juices (cup eq.)	0: Intake ≤ median<br>1: Intake > median
**Vegetables**	Total dark green, red and orange, starchy, and other vegetables; excludes legumes and potatoes (cup eq.)	0: Intake ≤ median<br>1: Intake > median
**Whole Grains**	Grains defined as whole grains and contain the entire grain kernel — the bran, germ, and endosperm (oz. eq.)	0: Intake ≤ median<br>1: Intake > median
**Legumes**	Beans and peas (cup eq.)	0: Intake ≤ median<br>1: Intake > median
**Nuts**	Peanuts, tree nuts, and seeds; excludes coconut (oz. eq.)	0: Intake ≤ median<br>1: Intake > median
**Fish**	Seafood (finfish, shellfish, and other seafood) – high and low n-3 fatty acids (oz. eq.)	0: Intake ≤ median<br>1: Intake > median
**SFA:MFA**	Ratio of Monosaturated Fatty Acids (g) to Saturated Fatty Acids (g)	0: Intake ≤ median<br>1: Intake > median
**Red Meat**	Beef, veal, pork, lamb, game meat, cured meat (frankfurters, sausages, corned beef, cured ham and luncheon meat that are made from beef, pork, or poultry) organ meat (from beef, veal, pork, lamb, game, and poultry) (oz. eq.)	0: Intake ≥ median<br>1: Intake < median
**Alcohol**	Total alcohol intake (g)	Females<br>0: Intake <5g or >15g<br>1: Intake ≥ 5g and ≤ 15g<br>Males<br>0: Intake <10g or >25g<br>1: Intake ≥ 10g and ≤ 25g
Abbreviations: eq (equivalents); g (gram); MFA:SFA (ratio of monosaturated fatty acid to saturated fatty acids); oz (ounce)		

Table S3.1: Components of Mediterranean Diet by Modified Fried Frailty Phenotype Definitions		Definition 1[1]				Definition 2[1]				Definition 3[1]			
	**Overall** N = 7,300	**Robust** N = 3,517 (51)	**Pre-frail** N = 3,173 (42)	**Frail** N = 610 (7.1)	**p-value[2]**	**Robust** N = 3,024 (44)	**Pre-frail** N = 3,537 (47)	**Frail** N = 739 (8.8)	**p-value[2]**	**Robust** N = 3,748 (54)	**Pre-frail** N = 3,063 (41)	**Frail** N = 489 (5.8)	**p-value[2]**
**Adherence to aMED Food Group[3]**													
Fruit	4,306 (58.8)	2,148 (60.5)	1,817 (57.3)	341 (56.0)	0.14	1,872 (61.1)	2,010 (56.6)	424 (59.5)	0.029	2,281 (60.2)	1,748 (57.4)	277 (56.5)	0.2
Vegetable	3,480 (52.0)	1,772 (54.7)	1,471 (50.3)	237 (42.5)	<0.001	1,531 (54.8)	1,646 (50.6)	303 (44.5)	0.001	1,863 (54.5)	1,423 (49.8)	194 (43.8)	0.002
Whole Grain	4,117 (59.8)	2,048 (62.1)	1,751 (58.1)	318 (54.1)	0.009	1,773 (62.4)	1,948 (58.1)	396 (56.4)	0.029	2,160 (61.8)	1,701 (58.0)	256 (54.6)	0.017
Legumes	1,452 (18.1)	735 (19.5)	609 (17.0)	108 (15.2)	0.076	613 (19.2)	709 (17.7)	130 (15.3)	0.3	775 (19.3)	595 (17.4)	82 (13.2)	0.079
Nuts	2,783 (45.2)	1,446 (48.0)	1,158 (43.9)	179 (32.4)	<0.001	1,242 (48.6)	1,313 (43.9)	228 (34.6)	<0.001	1,523 (48.0)	1,115 (43.4)	145 (32.3)	<0.001
Fish	1,387 (18.3)	723 (20.0)	572 (17.0)	92 (14.1)	0.016	623 (19.7)	650 (17.7)	114 (14.7)	0.084	750 (19.8)	561 (17.2)	76 (13.0)	0.022
Red Meat	3,910 (51.7)	1,848 (51.1)	1,740 (52.9)	322 (48.7)	0.4	1,596 (51.9)	1,920 (51.9)	394 (50.1)	0.9	1,958 (50.9)	1,694 (53.3)	258 (48.7)	0.3
MFA:SFA	4,017 (52.0)	1,967 (52.7)	1,760 (52.9)	290 (41.9)	0.006	1,688 (52.9)	1,963 (52.4)	366 (45.5)	0.087	2,098 (52.9)	1,686 (52.5)	233 (40.8)	0.004
Alcohol	475 (7.3)	289 (8.8)	164 (6.1)	22 (3.6)	<0.001	249 (8.9)	201 (6.4)	25 (3.7)	<0.001	306 (8.7)	150 (5.9)	19 (3.8)	<0.001
**Servings by Food Group**													
Fruit (cup)	1.0 (1.2)	1.1 (1.2)	1.0 (1.2)	1.0 (1.3)	0.075	1.0 (1.2)	1.1 (1.2)	1.0 (1.2)	0.026	1.0 (1.2)	1.1 (1.2)	1.0 (1.2)	0.2
Vegetable (cup)	1.2 (1.2)	1.3 (1.3)	1.1 (1.1)	1.0 (1.0)	<0.001	1.2 (1.2)	1.3 (1.3)	1.2 (1.1)	<0.001	1.2 (1.2)	1.3 (1.3)	1.1 (1.1)	<0.001
Whole Grain (cup)	1.0 (1.3)	1.1 (1.4)	1.0 (1.3)	0.8 (1.2)	0.019	1.0 (1.3)	1.1 (1.4)	1.0 (1.3)	0.075	1.0 (1.3)	1.1 (1.4)	1.0 (1.3)	0.040
Legumes (cup)	0.1 (0.3)	0.1 (0.3)	0.1 (0.3)	0.1 (0.3)	0.090	0.1 (0.3)	0.1 (0.3)	0.1 (0.3)	0.3	0.1 (0.3)	0.1 (0.3)	0.1 (0.3)	0.024
Nuts (cup)	0.8 (2.1)	0.9 (2.0)	0.8 (2.2)	0.6 (1.6)	<0.001	0.8 (2.1)	0.9 (2.0)	0.8 (2.2)	<0.001	0.8 (2.1)	0.9 (2.0)	0.8 (2.2)	<0.001
Fish (oz)	0.7 (2.0)	0.7 (2.1)	0.6 (2.0)	0.5 (1.9)	0.012	0.7 (2.0)	0.7 (2.1)	0.7 (2.0)	0.062	0.7 (2.0)	0.7 (2.1)	0.7 (2.0)	0.013
Red Meat (oz)	2.4 (2.7)	2.3 (2.5)	2.4 (2.8)	2.5 (2.7)	0.5	2.4 (2.7)	2.3 (2.6)	2.4 (2.7)	0.8	2.4 (2.7)	2.4 (2.5)	2.4 (2.8)	0.5
MFA:SFA	1.2 (0.4)	1.2 (0.4)	1.2 (0.4)	1.1 (0.5)	<0.001	1.2 (0.4)	1.2 (0.4)	1.2 (0.4)	0.036	1.2 (0.4)	1.2 (0.4)	1.2 (0.5)	<0.001
Alcohol (gm)	6.6 (17.7)	7.1 (16.6)	6.4 (19.0)	3.7 (16.9)	<0.001	6.6 (17.7)	7.3 (17.0)	6.4 (18.6)	<0.001	6.6 (17.7)	7.2 (18.1)	6.1 (17.3)	<0.001

1. Modified Fried Frailty Phenotype is based on 5 indicators (a) self-reported weakness: difficulty with lifting or carrying something as heavy as 10 pounds; (b) self-reported low physical activity: top quintile of minutes of sedentary activity; (c) self-reported exhaustion: feelings of tiredness or having little energy over the past two weeks for "more than half the days" or "nearly every day"; (d) self-reported slow walking speed: difficulty walking between rooms on the same floor; and (e) self-reported "weight loss" evaluated in three ways (i) Definition 1: unintentional weight loss ≥ 10lbs in the previous year; (ii) Definition 2: any weight loss ≥ 10lbs in the previous year; and (iii) Definition 3: low BMI defined as ≤18.5 kg/m^2; Robust: 0 indicators; Pre-frail: 1 to 2 indicators; Frail: 3 or more indicators; descriptive statistics presented as unweighted N (weighted %); Mean (SD);

2. Kruskal-Wallis rank-sum test for complex survey samples; chi-squared test with Rao & Scott's second-order correction;

3. Adherence to aMED food groups: intake ≥ median for fruit, vegetable, whole grain, legumes, nuts, and fish; intake < median for Red Meat and MFA:SFA; and within the sex-specific recommendations for alcohol;

Abbreviations: aMED (alternative Mediterranean Diet); BMI (body mass index); gm (gram); kg (kilogram); lb (pound); m (meter); MFA:SFA (Monosaturated Fat to Saturated Fat Ratio); N (number); oz (ounce); SD (standard deviation)

Table S3.2: Prevalence of Mediterranean Diet Adherence across Frailty Status

Variable		Definition 1[1]				Definition 2[1]				Definition 3[1]			
	**Overall** N = 7,300	**Robust** N = 3,517 (51)	**Pre-frail** N = 3,173 (42)	**Frail** N = 610 (7.1)	**p-value[2]**	**Robust** N = 3,024 (44)	**Pre-frail** N = 3,537 (47)	**Frail** N = 739 (8.8)	**p-value[2]**	**Robust** N = 3,748 (54)	**Pre-frail** N = 3,063 (41)	**Frail** N = 489 (5.8)	**p-value[2]**
**aMED Adherence Score[3]**	3.6 (1.6)	3.8 (1.6)	3.6 (1.6)	3.1 (1.5)	<0.001	3.8 (1.6)	3.6 (1.6)	3.2 (1.5)	<0.001	3.8 (1.6)	3.5 (1.6)	3.1 (1.5)	<0.001
**3-category[3]**					<0.001				<0.001				<0.001
Low	1,966 (25.5)	839 (22.6)	907 (27.0)	220 (38.0)		723 (22.3)	990 (26.7)	253 (34.9)		915 (22.8)	882 (27.3)	169 (38.1)	
Moderate	3,323 (44.7)	1,592 (44.5)	1,455 (45.0)	276 (44.1)		1,359 (44.0)	1,629 (45.5)	335 (43.6)		1,699 (44.4)	1,396 (45.0)	228 (44.3)	
High	2,011 (29.8)	1,086 (32.9)	811 (28.1)	114 (17.9)		942 (33.7)	918 (27.8)	151 (21.5)		1,134 (32.7)	785 (27.7)	92 (17.6)	
**4-category[3]**					<0.001				<0.001				<0.001
Low	1,966 (25.5)	839 (22.6)	907 (27.0)	220 (38.0)		723 (22.3)	990 (26.7)	253 (34.9)		915 (22.8)	882 (27.3)	169 (38.1)	
Low-Moderate	1,680 (22.6)	806 (22.1)	711 (22.4)	163 (26.8)		687 (21.9)	805 (22.9)	188 (24.5)		865 (22.1)	679 (22.5)	136 (26.5)	
High-Moderate	1,643 (22.1)	786 (22.4)	744 (22.6)	113 (17.2)		672 (22.1)	824 (22.6)	147 (19.2)		834 (22.3)	717 (22.5)	92 (17.8)	
High	2,011 (29.8)	1,086 (32.9)	811 (28.1)	114 (17.9)		942 (33.7)	918 (27.8)	151 (21.5)		1,134 (32.7)	785 (27.7)	92 (17.6)	
**Tertiles[3]**					<0.001				<0.001				<0.001
1st tertile	2,434 (30.6)	1,091 (28.2)	1,086 (31.5)	257 (42.6)		942 (28.0)	1,196 (31.5)	296 (38.9)		1,178 (28.3)	1,057 (31.8)	199 (42.8)	
2nd tertile	2,433 (33.4)	1,141 (32.7)	1,080 (33.8)	212 (35.8)		980 (32.4)	1,194 (34.0)	259 (34.6)		1,228 (32.8)	1,028 (33.8)	177 (36.1)	
3rd tertile	2,433 (36.0)	1,285 (39.1)	1,007 (34.8)	141 (21.7)		1,102 (39.5)	1,147 (34.5)	184 (26.5)		1,342 (38.9)	978 (34.4)	113 (21.0)	
**Quantiles[3]**					<0.001				<0.001				<0.001
1st quartile	1,825 (23.6)	786 (20.8)	833 (25.1)	206 (34.6)		678 (20.6)	913 (24.9)	234 (31.7)		856 (21.0)	811 (25.4)	158 (34.6)	
2nd quartile	1,825 (24.5)	861 (23.9)	787 (24.3)	177 (30.2)		734 (23.6)	884 (24.8)	207 (27.7)		926 (24.0)	752 (24.5)	147 (30.1)	
3rd quartile	1,825 (24.2)	882 (24.7)	816 (24.3)	127 (19.5)		760 (24.6)	904 (24.4)	161 (21.0)		934 (24.6)	789 (24.3)	102 (19.7)	
4th quartile	1,825 (27.7)	988 (30.6)	737 (26.3)	100 (15.7)		852 (31.2)	836 (25.9)	137 (19.7)		1,032 (30.4)	711 (25.8)	82 (15.6)	

1. Modified Fried Frailty Phenotype is based on 5 indicators (a) self-reported weakness: difficulty with lifting or carrying something as heavy as 10 pounds; (b) self-reported low physical activity: top quintile of minutes of sedentary activity; (c) self-reported exhaustion: feelings of tiredness or having little energy over the past two weeks for “more than half the days” or “nearly every day”; (d) self-reported slow walking speed: difficulty walking between rooms on the same floor; and (e) self-reported “weight loss” evaluated in three ways (i) Definition 1: unintentional weight loss ≥ 10lbs in the previous year; (ii) Definition 2: any weight loss ≥ 10lbs in the previous year; and (iii) Definition 3: low BMI defined as ≤18.5 kg/m^2; Robust: 0 indicators; Pre-frail: 1 to 2 indicators; Frail: 3 or more indicators; descriptive statistics presented as unweighted N (weighted %);
2. Kruskal-Wallis rank-sum test for complex survey samples; chi-squared test with Rao & Scott's second-order correction;
3. Continuous aMED adherence score (range 0 to 9) used for aMED adherence categories: (a) 3-category, Low 0-2, Moderate 3-4, High 5-9; (b) 4-category, Low 0-2, Low-Moderate 3, High-Moderate 4, and High 5-9; (c) tertiles; (d) quartiles

Abbreviations: aMED (alternative Mediterranean Diet); BMI (body mass index); gm (gram); kg (kilogram); lb (pound); m (meter); MFA:SFA (Monosaturated Fat to Saturated Fat Ratio); N (number); oz (ounce); SD (standard deviation)

Table S4.1: Univariate and Multivariate Logistic Regression of Frailty by Mediterranean Diet Score and Adherence (3-category)				
**Frail vs. Robust/Pre-frail[1]**	**aMED score[2]**	**Low[2]**	**Moderate[2]**	**High[2]**
**Definition 1**	*N = 610 (7.1%)*	*N = 220 (10.6%)*	*N = 276 (7.0%)*	*N = 114 (4.3%)*
Model 1	0.79 (0.73, 0.84)	Ref.	0.64 (0.50, 0.82)	0.38 (0.26, 0.54)
Model 2	0.81 (0.75, 0.87)	Ref.	0.64 (0.50, 0.82)	0.42 (0.29, 0.61)
Model 3	0.81 (0.75, 0.87)	Ref.	0.65 (0.51, 0.84)	0.43 (0.30, 0.62)
Model 4	0.84 (0.78, 0.91)	Ref.	0.71 (0.55, 0.92)	0.52 (0.36, 0.75)
**Definition 2**	*N = 739 (8.8%)*	*N = 253 (12.0%)*	*N = 335 (8.6%)*	*N = 151 (6.3%)*
Model 1	0.84 (0.79, 0.91)	Ref.	0.69 (0.54, 0.88)	0.50 (0.35, 0.69)
Model 2	0.86 (0.80, 0.93)	Ref.	0.69 (0.55, 0.87)	0.54 (0.38, 0.77)
Model 3	0.86 (0.80, 0.93)	Ref.	0.70 (0.55, 0.89)	0.55 (0.39, 0.78)
Model 4	0.91 (0.84, 0.98)	Ref.	0.76 (0.60, 0.97)	0.68 (0.48, 0.96)
**Definition 3**	*N = 489 (5.8%)*	*N = 169 (8.7%)*	*N = 228 (5.8%)*	*N = 92 (3.4%)*
Model 1	0.78 (0.73, 0.84)	Ref.	0.64 (0.49, 0.85)	0.37 (0.26, 0.53)
Model 2	0.80 (0.74, 0.86)	Ref.	0.64 (0.49, 0.84)	0.41 (0.28, 0.58)
Model 3	0.80 (0.74, 0.87)	Ref.	0.66 (0.50, 0.87)	0.42 (0.29, 0.60)
Model 4	0.84 (0.77, 0.90)	Ref.	0.72 (0.54, 0.96)	0.51 (0.36, 0.73)

1. Frail: 3 or more indicators; Robust/Pre-frail: 2 or less indicators; Modified Fried Frailty Phenotype is based on 5 indicators (a) self-reported weakness: difficulty with lifting or carrying something as heavy as 10 pounds; (b) self-reported low physical activity: top quintile of minutes of sedentary activity; (c) self-reported exhaustion: feelings of tiredness or having little energy over the past two weeks for "more than half the days" or "nearly every day"; (d) self-reported slow walking speed: difficulty walking between rooms on the same floor; and (e) self-reported "weight loss" evaluated in three ways (i) Definition 1: unintentional weight loss ≥ 10lbs in the previous year; (ii) Definition 2: any weight loss ≥ 10lbs in the previous year; and (iii) Definition 3: low BMI defined as ≤18.5 kg/m^2;
2. Continuous aMED adherence score (range 0 to 9) used for aMED adherence categories: Low 0-2 (referent), Moderate 3-4, and High 5-9; descriptive statistics for each definition presented as unweighted N (% weighted), Total unweighted N = 7,300; estimate presented as OR (95% CI);

Model 1: Unadjusted
Model 2: Adjusted for Sex, Age, Race, and Education
Model 3: Model 2 + smoking status
Model 4: Model 3 + polypharmacy and medical comorbidities score

Abbreviations: aMED (alternative Mediterranean Diet); BMI (body mass index); CI (confidence interval); kg (kilogram); lb (pound); m (meter); N (number); OR (odds ratio)

**Table S4.2: Univariate and Multivariate Logistic Regression of Frailty by Mediterranean Diet Score and Adherence (4-category)**					
**Frail vs. Robust/Pre-frail[1]**	**aMED score[2]**	**Low[2]**	**Low-Moderate[2]**	**High-Moderate[2]**	**High[2]**
**Definition 1**	*N = 610 (7.1%)*	*N = 220 (10.6%)*	*N = 163 (8.5%)*	*N = 113 (5.6%)*	*N = 114 (4.3%)*
Model 1	0.79 (0.73, 0.84)	Ref.	0.78 (0.56, 1.08)	0.50 (0.39, 0.64)	0.38 (0.26, 0.54)
Model 2	0.81 (0.75, 0.87)	Ref.	0.78 (0.57, 1.08)	0.50 (0.38, 0.64)	0.42 (0.29, 0.60)
Model 3	0.81 (0.75, 0.87)	Ref.	0.80 (0.58, 1.11)	0.51 (0.39, 0.66)	0.43 (0.30, 0.62)
Model 4	0.84 (0.78, 0.91)	Ref.	0.88 (0.62, 1.24)	0.55 (0.42, 0.71)	0.52 (0.36, 0.75)
**Definition 2**	*N = 739 (8.8%)*	*N = 253 (12.0%)*	*189 (9.5%)*	*N = 147 75.6%)*	*N = 151 (6.3%)*
Model 1	0.84 (0.79, 0.91)	Ref.	0.77 (0.56, 1.07)	0.60 (0.48, 0.76)	0.50 (0.35, 0.69)
Model 2	0.86 (0.80, 0.93)	Ref.	0.77 (0.56, 1.06)	0.61 (0.48, 0.77)	0.54 (0.38, 0.76)
Model 3	0.86 (0.80, 0.93)	Ref.	0.78 (0.57, 1.08)	0.62 (0.49, 0.78)	0.55 (0.39, 0.78)
Model 4	0.91 (0.84, 0.98)	Ref.	0.85 (0.61, 1.20)	0.67 (0.52, 0.86)	0.68 (0.48, 0.96)
**Definition 3**	*N = 489 (5.8%)*	*N = 169 (8.7%)*	*136 (6.8%)*	*N = 92 (4.7%)*	*N = 92 (3.4%)*
Model 1	0.78 (0.73, 0.84)	Ref.	0.77 (0.54, 1.11)	0.52 (0.39, 0.69)	0.37 (0.26, 0.53)
Model 2	0.80 (0.74, 0.86)	Ref.	0.77 (0.54, 1.10)	0.51 (0.38, 0.69)	0.40 (0.28, 0.58)
Model 3	0.80 (0.74, 0.87)	Ref.	0.79 (0.55, 1.13)	0.52 (0.39, 0.71)	0.41 (0.29, 0.59)
Model 4	0.84 (0.77, 0.90)	Ref.	0.87 (0.59, 1.28)	0.57 (0.43, 0.78)	0.51 (0.36, 0.73)

1. Frail: 3 or more indicators; Robust/Pre-frail: 2 or less indicators; Modified Fried Frailty Phenotype is based on 5 indicators (a) self-reported weakness: difficulty with lifting or carrying something as heavy as 10 pounds; (b) self-reported low physical activity: top quintile of minutes of sedentary activity; (c) self-reported exhaustion: feelings of tiredness or having little energy over the past two weeks for "more than half the days" or "nearly every day"; (d) self-reported slow walking speed: difficulty walking between rooms on the same floor; and (e) self-reported "weight loss" evaluated in three ways (i) Definition 1: unintentional weight loss ≥ 10lbs in the previous year; (ii) Definition 2: any weight loss ≥ 10lbs in the previous year; and (iii) Definition 3: low BMI defined as ≤18.5 kg/m^2;
2. Continuous aMED adherence score (range 0 to 9) used for aMED adherence categories: Low 0-2 (referent), Low-Moderate 3, High-Moderate 4, and High 5-9; descriptive statistics for each definition presented as unweighted N (% weighted), Total unweighted N = 7,300; estimate presented as OR (95% CI);

Model 1: Unadjusted
Model 2: Adjusted for Sex, Age, Race, and Education
Model 3: Model 2 + smoking status
Model 4: Model 3 + polypharmacy and medical comorbidities score

Abbreviations: aMED (alternative Mediterranean Diet); BMI (body mass index); CI (confidence interval); kg (kilogram); lb (pound); m (meter); N (number); OR (odds ratio)

Table S4.3: Univariate and Multivariate Logistic Regression of Frailty by Mediterranean Diet Score and Adherence (tertiles)				
**Frail vs. Robust/Pre-frail[1]**	**aMED score[2]**	**Low[2]**	**Moderate[2]**	**High[2]**
**Definition 1**	*N = 610 (7.1%)*	*N = 257 (9.9%)*	*N = 212 (7.6%)*	*N = 141 (4.3%)*
Model 1	0.79 (0.73, 0.84)	Ref.	0.75 (0.57, 0.98)	0.41 (0.31, 0.54)
Model 2	0.81 (0.75, 0.87)	Ref.	0.76 (0.59, 0.99)	0.45 (0.33, 0.60)
Model 3	0.81 (0.75, 0.87)	Ref.	0.78 (0.60, 1.02)	0.46 (0.34, 0.61)
Model 4	0.84 (0.78, 0.91)	Ref.	0.83 (0.63, 1.09)	0.52 (0.39, 0.68)
**Definition 2**	*N = 739 (8.8%)*	*N = 296 (11.2%)*	*N = 259 (9.1%)*	*N = 184 (6.5%)*
Model 1	0.84 (0.79, 0.91)	Ref.	0.80 (0.61, 1.04)	0.55 (0.41, 0.73)
Model 2	0.86 (0.80, 0.93)	Ref.	0.81 (0.63, 1.05)	0.59 (0.44, 0.80)
Model 3	0.86 (0.80, 0.93)	Ref.	0.83 (0.64, 1.07)	0.61 (0.45, 0.82)
Model 4	0.91 (0.84, 0.98)	Ref.	0.88 (0.67, 1.15)	0.70 (0.52, 0.94)
**Definition 3**	*N = 489 (5.8%)*	*N = 199 (8.1%)*	*N = 177 (6.3%)*	*N = 113 (3.4%)*
Model 1	0.78 (0.73, 0.84)	Ref.	0.76 90.57, 1.01)	0.40 (0.30, 0.53)
Model 2	0.80 (0.74, 0.86)	Ref.	0.76 (0.57, 1.01)	0.42 (0.31, 0.57)
Model 3	0.80 (0.74, 0.87)	Ref.	0.78 (0.59, 1.04)	0.44 (0.32, 0.59)
Model 4	0.84 (0.77, 0.90)	Ref.	0.83 (0.62, 1.12)	0.50 (0.37, 0.66)

1. Frail: 3 or more indicators; Robust/Pre-frail: 2 or less indicators; Modified Fried Frailty Phenotype is based on 5 indicators (a) self-reported weakness: difficulty with lifting or carrying something as heavy as 10 pounds; (b) self-reported low physical activity: top quintile of minutes of sedentary activity; (c) self-reported exhaustion: feelings of tiredness or having little energy over the past two weeks for “more than half the days” or “nearly every day”; (d) self-reported slow walking speed: difficulty walking between rooms on the same floor; and (e) self-reported “weight loss” evaluated in three ways (i) Definition 1: unintentional weight loss ≥ 10lbs in the previous year; (ii) Definition 2: any weight loss ≥ 10lbs in the previous year; and (iii) Definition 3: low BMI defined as ≤18.5 kg/m^2;
2. Continuous aMED adherence score (range 0 to 9) used for aMED adherence categories: Low 1st tertile (referent), Moderate 2nd tertile, and High 3rd tertile; descriptive statistics for each definition presented as unweighted N (% weighted), Total unweighted N = 7,300; estimate presented as OR (95% CI);

Model 1: Unadjusted
Model 2: Adjusted for Sex, Age, Race, and Education
Model 3: Model 2 + smoking status
Model 4: Model 3 + polypharmacy and medical comorbidities score

Abbreviations: aMED (alternative Mediterranean Diet); BMI (body mass index); CI (confidence interval); kg (kilogram); lb (pound); m (meter); N (number); OR (odds ratio)

Table S4.4: Univariate and Multivariate Logistic Regression of Frailty by Mediterranean Diet Score and Adherence (quartiles)

Frail vs. Robust/Pre-frail[1]	aMED score[2]	Low[2]	Low-Moderate[2]	High-Moderate[2]	High[2]
**Definition 1**	*N = 610 (7.1%)*	*N = 206 (10.5%)*	*N = 177 (8.8%)*	*N = 127 (5.7%)*	*N = 100 (4.1%)*
Model 1	0.79 (0.73, 0.84)	Ref.	0.82 (0.59, 1.15)	0.52 (0.40, 0.68)	0.36 (0.25, 0.51)
Model 2	0.81 (0.75, 0.87)	Ref.	0.83 (0.60, 1.16)	0.53 (0.40, 0.69)	0.40 (0.28, 0.58)
Model 3	0.81 (0.75, 0.87)	Ref.	0.85 (0.61, 1.19)	0.54 (0.41, 0.71)	0.41 (0.29, 0.59)
Model 4	0.84 (0.78, 0.91)	Ref.	0.87 (0.62, 1.24)	0.57 (0.43, 0.74)	0.49 (0.34, 0.70)
**Definition 2**	*N = 739 (8.8%)*	*N = 234 (11.8%)*	*N = 207 (9.9%)*	*N = 161 (7.6%)*	*N = 137 (6.3%*
Model 1	0.84 (0.79, 0.91)	Ref.	0.82 (0.59, 1.15)	0.62 (0.49, 0.78)	0.50 (0.36, 0.70)
Model 2	0.86 (0.80, 0.93)	Ref.	0.83 (0.60, 1.14)	0.62 (0.49, 0.79)	0.54 (0.38, 0.77)
Model 3	0.86 (0.80, 0.93)	Ref.	0.84 (0.61, 1.17)	0.64 (0.50, 0.81)	0.56 (0.39, 0.79)
Model 4	0.91 (0.84, 0.98)	Ref.	0.86 (0.61, 1.22)	0.67 (0.53, 0.86)	0.68 (0.48, 0.96)
**Definition 3**	*N = 489 (5.8%)*	*N = 158 (8.5%)*	*N = 147 (7.1%)*	*N = 102 (4.7%)*	*N = 82 (3.3%)*
Model 1	0.78 (0.73, 0.84)	Ref.	0.82 (0.57, 1.20)	0.53 (0.40, 0.71)	0.36 (0.26, 0.52)
Model 2	0.80 (0.74, 0.86)	Ref.	0.83 (0.57, 1.20)	0.53 (0.40, 0.71)	0.40 (0.28, 0.57)
Model 3	0.80 (0.74, 0.87)	Ref.	0.85 (0.58, 1.24)	0.55 (0.41, 0.74)	0.41 (0.28, 0.58)
Model 4	0.84 (0.77, 0.90)	Ref.	0.87 (0.58, 1.29)	0.58 (0.43, 0.79)	0.49 (0.34, 0.71)

1. Frail: 3 or more indicators; Robust/Pre-frail: 2 or less indicators; Modified Fried Frailty Phenotype is based on 5 indicators (a) self-reported weakness: difficulty with lifting or carrying something as heavy as 10 pounds; (b) self-reported low physical activity: top quintile of minutes of sedentary activity; (c) self-reported exhaustion: feelings of tiredness or having little energy over the past two weeks for "more than half the days" or "nearly every day"; (d) self-reported slow walking speed: difficulty walking between rooms on the same floor; and (e) self-reported "weight loss" evaluated in three ways (i) Definition 1: unintentional weight loss ≥ 10lbs in the previous year; (ii) Definition 2: any weight loss ≥ 10lbs in the previous year; and (iii) Definition 3: low BMI defined as ≤18.5 kg/m^2;
2. Continuous aMED adherence score (range 0 to 9) used for aMED adherence categories: Low 1st quartile (referent), Low-Moderate 2nd quartile, High-Moderate 3rd quartile, and High 4th quartile; descriptive statistics for each definition presented as unweighted N (% weighted), Total unweighted N = 7,300; estimate presented as OR (95% CI);

Model 1: Unadjusted
Model 2: Adjusted for Sex, Age, Race, and Education
Model 3: Model 2 + smoking status
Model 4: Model 3 + polypharmacy and medical comorbidities score

Abbreviations: aMED (alternative Mediterranean Diet); BMI (body mass index); CI (confidence interval); kg (kilogram); lb (pound); m (meter); N (number); OR (odds ratio)

Table S5.1: Univariate and Multivariate Logistic Regression of Frailty by Mediterranean Diet Score and Adherence – Sensitivity Analysis (3-category)				
**Pre-frail/Frail vs. Robust[1]**	**aMED score[2]**	**Low[2]**	**Moderate[2]**	**High[2]**
**Definition 1**	*N = 3,783 (49%)*	*N = 1,127 (54.9%)*	*N = 1,731 (49.2%)*	*N = 925 (43.7%)*
Model 1	0.89 (0.85, 0.94)	Ref.	0.79 (0.68, 0.93)	0.64 (0.53, 0.77)
Model 2	0.90 (0.85, 0.95)	Ref.	0.80 (0.68, 0.93)	0.66 (0.54, 0.80)
Model 3	0.90 (0.86, 0.95)	Ref.	0.80 (0.69, 0.94)	0.66 (0.54, 0.81)
Model 4	0.93 (0.88, 0.98)	Ref.	0.83 (0.71, 0.97)	0.74 (0.61, 0.91)
**Definition 2**	*N = 4,276 (55.7%)*	*N = 1,243 (61.2%)*	*N = 1,964 (56.4%)*	*N = 1,069 (50%)*
Model 1	0.89 (0.85, 0.94)	Ref.	0.82 (0.71, 0.94)	0.63 (0.52, 0.77)
Model 2	0.90 (0.86, 0.95)	Ref.	0.83 (0.71, 0.96)	0.66 (0.54, 0.82)
Model 3	0.90 (0.86, 0.96)	Ref.	0.83 (0.72, 0.97)	0.67 (0.54, 0.83)
Model 4	0.93 (0.88, 0.98)	Ref.	0.86 (0.74, 1.01)	0.75 (0.60, 0.93)
**Definition 3**	*N = 3,552 (46.3%)*	*N = 1,051 (52%)*	*N = 1,624 (46.7%)*	*N = 877 (41.1%)*
Model 1	0.90 (0.85, 0.94)	Ref.	0.81 (0.69, 0.94)	0.64 (0.53, 0.78)
Model 2	0.90 (0.85, 0.95)	Ref.	0.80 (0.69, 0.94)	0.65 (0.52, 0.81)
Model 3	0.90 (0.85, 0.95)	Ref.	0.81 (0.69, 0.95)	0.66 (0.53, 0.81)
Model 4	0.93 (0.88, 0.98)	Ref.	0.84 (0.71, 0.99)	0.74 (0.59, 0.92)

1. Pre-frail/Frail: 1 or more indicators; Robust: no indicators; Modified Fried Frailty Phenotype is based on 5 indicators (a) self-reported weakness: difficulty with lifting or carrying something as heavy as 10 pounds; (b) self-reported low physical activity: top quintile of minutes of sedentary activity; (c) self-reported exhaustion: feelings of tiredness or having little energy over the past two weeks for “more than half the days” or “nearly every day”; (d) self-reported slow walking speed: difficulty walking between rooms on the same floor; and (e) self-reported “weight loss” evaluated in three ways (i) Definition 1: unintentional weight loss ≥ 10lbs in the previous year; (ii) Definition 2: any weight loss ≥ 10lbs in the previous year; and (iii) Definition 3: low BMI defined as ≤18.5 kg/m^2;
2. Continuous aMED adherence score (range 0 to 9) used for aMED adherence categories: Low 1st quartile (referent), Low-Moderate 2nd quartile, High-Moderate 3rd quartile, and High 4th quartile; descriptive statistics for each definition presented as unweighted N (% weighted), Total unweighted N = 7,300; estimate presented as OR (95% CI);

Model 1: Unadjusted
Model 2: Adjusted for Sex, Age, Race, and Education
Model 3: Model 2 + smoking status
Model 4: Model 3 + polypharmacy and medical comorbidities score

Abbreviations: aMED (alternative Mediterranean Diet); BMI (body mass index); CI (confidence interval); kg (kilogram); lb (pound); m (meter); N (number); OR (odds ratio)

Table S5.2: Univariate and Multivariate Logistic Regression of Frailty by Mediterranean Diet Score and Adherence – Sensitivity Analysis (4-category)

Pre-frail/Frail vs. Robust[1]	aMED score[2]	Low[2]	Low-Moderate[2]	High-Moderate[2]	High[2]
**Definition 1**	*N = 3,783 (49%)*	*N = 1,127 (54.9%)*	*N = 874 (50%)*	*N = 857 (48.4%)*	*N = 925 (43.7%)*
Model 1	0.89 (0.85, 0.94)	Ref.	0.82 (0.69, 0.98)	0.77 (0.63, 0.93)	0.64 (0.53, 0.77)
Model 2	0.90 (0.85, 0.95)	Ref.	0.82 (0.69, 0.97)	0.77 (0.63, 0.94)	0.66 (0.54, 0.80)
Model 3	0.90 (0.86, 0.95)	Ref.	0.83 (0.70, 0.98)	0.78 (0.64, 0.95)	0.66 (0.54, 0.81)
Model 4	0.93 (0.88, 0.98)	Ref.	0.85 (0.72, 1.01)	0.81 (0.66, 0.99)	0.74 (0.61, 0.91)
**Definition 2**	*N = 4,276 (55.7%)*	*N = 1,243 (61.2%)*	*N = 993 (57.1%)*	*N = 971 (55.7%)*	*N = 1,069 (50%)*
Model 1	0.89 (0.85, 0.94)	Ref.	0.84 (0.71, 1.00)	0.80 (0.67, 0.95)	0.63 (0.52, 0.77)
Model 2	0.90 (0.86, 0.95)	Ref.	0.84 (0.71, 1.00)	0.81 (0.68, 0.97)	0.66 (0.54, 0.82)
Model 3	0.90 (0.86, 0.96)	Ref.	0.85 (0.72, 1.01)	0.82 (0.68, 0.98)	0.67 (0.54, 0.83)
Model 4	0.93 (0.88, 0.98)	Ref.	0.88 (0.74, 1.05)	0.85 (0.74, 1.05)	0.75 (0.60, 0.93)
**Definition 3**	*N = 3,552 (46.3%)*	*N = 1,051 (52%)*	*N = 1,051 (47.3%)*	*N = 809 (45.9%)*	*N = 877 (41.1%)*
Model 1	0.90 (0.85, 0.94)	Ref.	0.83 (0.70, 0.99)	0.78 (0.65, 0.94)	0.64 (0.53, 0.78)
Model 2	0.90 (0.85, 0.95)	Ref.	0.82 (0.69, 0.98)	0.78 (0.65, 0.94)	0.65 (0.52, 0.81)
Model 3	0.90 (0.85, 0.95)	Ref.	0.83 (0.70, 0.99)	0.79 (0.65, 0.95)	0.65 (0.53, 0.81)
Model 4	0.93 (0.88, 0.98)	Ref.	0.86 (0.71, 1.03)	0.82 (0.67, 0.99)	0.74 (0.59, 0.92)

1. Pre-frail/Frail: 1 or more indicators; Robust: no indicators; Modified Fried Frailty Phenotype is based on 5 indicators (a) self-reported weakness: difficulty with lifting or carrying something as heavy as 10 pounds; (b) self-reported low physical activity: top quintile of minutes of sedentary activity; (c) self-reported exhaustion: feelings of tiredness or having little energy over the past two weeks for "more than half the days" or "nearly every day"; (d) self-reported slow walking speed: difficulty walking between rooms on the same floor; and (e) self-reported "weight loss" evaluated in three ways (i) Definition 1: unintentional weight loss ≥ 10lbs in the previous year; (ii) Definition 2: any weight loss ≥ 10lbs in the previous year; and (iii) Definition 3: low BMI defined as ≤18.5 kg/m^2;
2. Continuous aMED adherence score (range 0 to 9) used for aMED adherence categories: Low 0-2 (referent), Low-Moderate 3, High-Moderate 4, and High 5-9; descriptive statistics for each definition presented as unweighted N (% weighted), Total unweighted N = 7,300; estimate presented as OR (95% CI);

Model 1: Unadjusted
Model 2: Adjusted for Sex, Age, Race, and Education
Model 3: Model 2 + smoking status
Model 4: Model 3 + polypharmacy and medical comorbidities score

Abbreviations: aMED (alternative Mediterranean Diet); BMI (body mass index); CI (confidence interval); kg (kilogram); lb (pound); m (meter); N (number); OR (odds ratio)

Table S5.3: Univariate and Multivariate Logistic Regression of Frailty by Mediterranean Diet Score and Adherence – Sensitivity Analysis (tertiles)				
**Pre-frail/Frail vs. Robust[1]**	**aMED score[2]**	**Low[2]**	**Moderate[2]**	**High[2]**
**Definition 1**	*N = 3,783 (49%)*	*N = 1,343 (53%)*	*N = 1,292 (50%)*	*N = 1,148 (44.7%)*
Model 1	0.89 (0.85, 0.94)	Ref.	0.89 (0.76, 1.04)	0.72 (0.61, 0.85)
Model 2	0.90 (0.85, 0.95)	Ref.	0.90 (0.76, 1.06)	0.75 (0.62, 0.89)
Model 3	0.90 (0.86, 0.95)	Ref.	0.91 (0.77, 1.07)	0.75 (0.63, 0.90)
Model 4	0.93 (0.88, 0.98)	Ref.	0.94 (0.79, 1.11)	0.83 (0.68, 1.00)
**Definition 2**	*N = 4,276 (55.7%)*	*N = 1,492 (59.5%)*	*N = 1,453 (57%)*	*N = 1,331 (51.5%)*
Model 1	0.89 (0.85, 0.94)	Ref.	0.90 (0.78, 1.05)	0.72 (0.61, 0.86)
Model 2	0.90 (0.86, 0.95)	Ref.	0.92 (0.79, 1.08)	0.76 (0.63, 0.92)
Model 3	0.90 (0.86, 0.96)	Ref.	0.93 (0.79, 1.09)	0.77 (0.64, 0.93)
Model 4	0.93 (0.88, 0.98)	Ref.	0.96 (0.82, 1.13)	0.84 (0.69, 1.03)
**Definition 3**	*N = 3,552 (46.3%)*	*N = 1,256 (51.3%)*	*N = 1,205 (47.3%)*	*N = 1,091 (42.1%)*
Model 1	0.90 (0.85, 0.94)	Ref.	0.89 (0.76, 1.03)	0.72 (0.61, 0.85)
Model 2	0.90 (0.85, 0.95)	Ref.	0.89 (0.76, 1.05)	0.73 (0.61, 0.88)
Model 3	0.90 (0.85, 0.95)	Ref.	0.90 (0.77, 1.06)	0.74 (0.61, 0.89)
Model 4	0.93 (0.88, 0.98)	Ref.	0.93 (0.78, 1.10)	0.81 (0.67, 0.99)

1. Pre-frail/Frail: 1 or more indicators; Robust: no indicators; Modified Fried Frailty Phenotype is based on 5 indicators (a) self-reported weakness: difficulty with lifting or carrying something as heavy as 10 pounds; (b) self-reported low physical activity: top quintile of minutes of sedentary activity; (c) self-reported exhaustion: feelings of tiredness or having little energy over the past two weeks for "more than half the days" or "nearly every day"; (d) self-reported slow walking speed: difficulty walking between rooms on the same floor; and (e) self-reported "weight loss" evaluated in three ways (i) Definition 1: unintentional weight loss ≥ 10lbs in the previous year; (ii) Definition 2: any weight loss ≥ 10lbs in the previous year; and (iii) Definition 3: low BMI defined as ≤18.5 kg/m^2;
2. Continuous aMED adherence score (range 0 to 9) used for aMED adherence categories: Low 1st tertile (referent), Moderate 2nd tertile, and High 3rd tertile; descriptive statistics for each definition presented as unweighted N (% weighted), Total unweighted N = 7,300; estimate presented as OR (95% CI);

Model 1: Unadjusted
Model 2: Adjusted for Sex, Age, Race, and Education
Model 3: Model 2 + smoking status
Model 4: Model 3 + polypharmacy and medical comorbidities score

Abbreviations: aMED (alternative Mediterranean Diet); BMI (body mass index); CI (confidence interval); kg (kilogram); lb (pound); m (meter); N (number); OR (odds ratio)

Table S5.4: Univariate and Multivariate Logistic Regression of Frailty by Mediterranean Diet Score and Adherence – Sensitivity Analysis (quartiles)					
**Pre-frail/Frail vs. Robust[1]**	**aMED score[2]**	**Low[2]**	**Low-Moderate[2]**	**High-Moderate[2]**	**High[2]**
**Definition 1**	*N = 3,783 (49%)*	*N = 1,039 (55.1%)*	*N = 964 (50.3%)*	*N = 943 (47.8%)*	*N = 837 (43.8%)*
Model 1	0.89 (0.85, 0.94)	Ref.	0.82 (0.69, 0.98)	0.75 (0.61, 0.91)	0.63 (0.52, 0.77)
Model 2	0.90 (0.85, 0.95)	Ref.	0.82 (0.70, 0.98)	0.76 (0.62, 0.93)	0.66 (0.53, 0.81)
Model 3	0.90 (0.86, 0.95)	Ref.	0.83 (0.70, 0.99)	0.76 (0.62, 0.93)	0.66 (0.54, 0.82)
Model 4	0.93 (0.88, 0.98)	Ref.	0.85 (0.71, 1.01)	0.79 (0.64, 0.97)	0.75 (0.60, 0.92)
**Definition 2**	*N = 4,276 (55.7%)*	*N = 1,147 (52.2%)*	*N = 1,091 (57.4%)*	*N = 1,065 (54.9%)*	*N = 973 (50.3%)*
Model 1	0.89 (0.85, 0.94)	Ref.	0.85 (0.73, 1.00)	0.77 (0.64, 0.92)	0.64 (0.52, 0.78)
Model 2	0.90 (0.86, 0.95)	Ref.	0.86 (0.73, 1.01)	0/79 (0.66, 0.95)	0.67 (0.54, 0.83)
Model 3	0.90 (0.86, 0.96)	Ref.	0.87 (0.74, 1.02)	0.80 (0.66, 0.96)	0.68 (0.54, 0.84)
Model 4	0.93 (0.88, 0.98)	Ref.	0.89 (0.75, 1.05)	0.82 (0.68, 0.99)	0.78 (0.60, 0.96)
**Definition 3**	*N = 3,552 (46.3%)*	*N = 969 (52.2%)*	*N = 899 (47.5%)*	*N = 891 (45.5%)*	*N = 793 (41.1%)*
Model 1	0.90 (0.85, 0.94)	Ref.	0.83 (0.70, 0.98)	0.76 (0.63, 0.93)	0.64 (0.52, 0.78)
Model 2	0.90 (0.85, 0.95)	Ref.	0.83 (0.70, 0.98)	0.76 (0.63, 0.93)	0.64 (0.51, 0.80)
Model 3	0.90 (0.85, 0.95)	Ref.	0.84 (0.71, 0.99)	0.77 (0.63, 0.94)	0.65 (0.52, 0.81)
Model 4	0.93 (0.88, 0.98)	Ref.	0.85 (0.71, 1.01)	0.80 (0.65, 0.97)	0.73 (0.58, 0.92)

1. Pre-frail/Frail: 1 or more indicators; Robust: no indicators; Modified Fried Frailty Phenotype is based on 5 indicators (a) self-reported weakness: difficulty with lifting or carrying something as heavy as 10 pounds; (b) self-reported low physical activity: top quintile of minutes of sedentary activity; (c) self-reported exhaustion: feelings of tiredness or having little energy over the past two weeks for “more than half the days” or “nearly every day”; (d) self-reported slow walking speed: difficulty walking between rooms on the same floor; and (e) self-reported “weight loss” evaluated in three ways (i) Definition 1: unintentional weight loss ≥ 10lbs in the previous year; (ii) Definition 2: any weight loss ≥ 10lbs in the previous year; and (iii) Definition 3: low BMI defined as ≤18.5 kg/m^2;
2. Continuous aMED adherence score (range 0 to 9) used for aMED adherence categories: Low 1st quartile (referent), Low-Moderate 2nd quartile, High-Moderate 3rd quartile, and High 4th quartile; descriptive statistics for each definition presented as unweighted N (% weighted), Total unweighted N = 7,300; estimate presented as OR (95% CI);

Model 1: Unadjusted
Model 2: Adjusted for Sex, Age, Race, and Education
Model 3: Model 2 + smoking status
Model 4: Model 3 + polypharmacy and medical comorbidities score

Abbreviations: aMED (alternative Mediterranean Diet); BMI (body mass index); CI (confidence interval); kg (kilogram); lb (pound); m (meter); N (number); OR (odds ratio)
